# Antibiotic Resistance Profile of Enterovirulent *E. coli* Isolates Harboring Broad-Spectrum Beta-Lactamase Genes in Cancer Patients at the Laquintinie Hospital in Douala, Littoral Region, Cameroon

**DOI:** 10.1155/ijm/4224807

**Published:** 2025-01-09

**Authors:** Michael F. Kengne, Armelle T. Mbaveng, Wiliane J. T. Marbou, Ousenu Karimo, Ballue S. T. Dadjo, Delano G. T. Fonjou, Ornella D. Tsobeng, Victor Kuete

**Affiliations:** Department of Biochemistry, Faculty of Science, Université de Dschang, Dschang, Cameroon

**Keywords:** antimicrobial resistance, cancer patients, enterovirulent *E. coli*, ESBL

## Abstract

Cases of antibiotic-resistant *Escherichia coli* (*E. coli*) infections are becoming increasingly frequent and represent a major threat to our ability to treat cancer patients. The emergence of antimicrobial resistance threatens the treatment of *E. coli* infections. In this study, the antimicrobial profiles, virulent genes, and the frequency of extended-spectrum beta-lactamase (ESBL) gene carriage in fecal *E. coli* isolates from cancer patients at the Laquintinie Hospital in Douala (Cameroon) were determined. 507 participants were recruited from October 2021 to March 2023, of whom 307 (60.55%) had cancer and 200 (39.45%) did not. Two hundred and two *E. coli* were isolated from fecal samples of one hundred and fifteen cancer patients and 47 (87) noncancer patients using EMB LEVINE agar. The antimicrobial resistance profile of the isolates was determined using the Kirby–Bauer disk diffusion method. Virulence and resistance genes were detected by simplex polymerase chain reaction (PCR). *E. coli* showed significant rates of resistance to amoxicillin, cefotaxime, ceftazidime, piperacillin, tetracycline, and ciprofloxacin in cancer patients compared to noncancer patients. The rate of multidrug resistance (MDR) was significantly (*p* < 0.05) higher in cancer patients than in noncancer patients. Fifty-five enterovirulent *E. coli* were identified, of which 24 (43.63%) were EPEC, 13 (23.63%) were EAEC, 6 (10.90%) were ETEC, 10 (18.18%) were STEC, and 2 (3.63%) were EIEC. The frequency of beta-lactamase genes in the 55 ESBL-producing enterovirulent *E. coli* isolates was determined, and 94.54% harbored at least one ESBL gene, distributed as follows: 80.00% for *bla*_TEM_, 67.27% for *bla*_CTX−M_, 24.63 for *bla*_OXA_, and 36.36% for *bla*_SHV_ genes. Several associations were observed between virulence factors, resistance genes, and the antimicrobial resistance phenotype. This study revealed the real existence of fecal carriage of ESBL-producing enterovirulent *E. coli* isolates from cancer patients with a high rate of MDR in the latter.

## 1. Introduction

The intestinal microbiota, or simply the intestinal flora, are the microorganisms, including bacteria, archaea, fungi, and viruses, that live in the digestive tract of humans and animals [1]. The intestinal microbiota have several impacts on the body, including consequences for colonization, resistance to pathogens, maintenance of the intestinal epithelium, metabolism of food and pharmaceutical compounds, and control of the immune system [2]. Bacterial families called Enterobacteriaceae colonize the intestine. Enterobacteriaceae include *Escherichia coli* (*E. coli*), which is part of the normal enteric bacterial flora of humans and animals but can develop pathogenic mechanisms, causing enteric diarrheal infections in humans [3]. *E. coli* can also cause extremely severe clinical manifestations leading to death, and its pathogenicity varies depending on the microbial species, the condition of the host, its association with other microorganisms, or the antibiotic therapy received [4]. On the other hand, any virulent *E. coli* or major reservoir of extended-spectrum beta-lactamase (ESBL) genes, which confer resistance to several *β*-lactam antibiotics, can be pathogenic and harmful to its host [5]. There are six intestinal *E. coli* pathotypes described in the literature that are associated with acute diarrheal disease based on the set of virulence factors they express, and these include Shiga toxin–producing *E. coli* (STEC), enteropathogenic *E. coli* (EPEC), enteroaggregative *E. coli* (EAEC), enterotoxigenic *E. coli* (ETEC), diffuse adherent *E. coli* (DAEC), and enteroinvasive *E. coli (*EIEC) [6]. It should be noted that diarrhea is a common reason in oncology wards for delaying or reducing the intensity of chemotherapy, ultimately leading to increased mortality [7]. Clinicians are often faced with the challenge of determining whether diarrhea in a cancer patient is due to infection with an enteropathogenic or epithelial cell loss associated with chemotherapy. While EPEC is considered an important cause of pediatric diarrhea in developing countries, its role as an enteric pathogen in cancer patients is less clear. The categorization of *E. coli* as EPEC is based on the presence of the plasmid-encoded bundle-forming pilus (*bfpA*) gene, and *E. coli* is classified as ETEC based on the presence of *LT* genes. The *AggR* plasmid-encoded gene is used to classify *EAEC E. coli* isolates, and *E. coli* are classified as STEC and EIEC based on the presence of *Stx* and *IpaH* genes, respectively [6]. Infection with *E. coli* can manifest itself through symptoms such as abdominal pain, nausea, an urgent need to have a bowel movement but without result, and vomiting accompanied by little or no fever [3]. Diarrhea is a manifestation that diminishes the quality of life of cancer patients, affecting more than half of patients undergoing certain types of chemotherapy and around 10% of hospitalized patients [8]. Diarrheal diseases are one of the main causes of morbidity and mortality worldwide, particularly in developing countries [9]. Diarrheal diseases are a real public health burden in Cameroon, with the Center for Disease Control and Prevention (CDC) stating in 2018 that they are the fifth leading cause of death in Cameroon [10, 11]. Antimicrobial drugs are substances used to treat infectious diseases of bacterial origin [12]. The uncontrolled use of these antimicrobial drugs is closely linked to the selection of antimicrobial resistance (AMR) bacteria, and the intestinal flora is a reservoir of AMR genes, since ESBL *E. coli*, for example, can appear in the human or animal intestine following the use of antibiotics [13, 14]. The increase and spread of bacterial resistance to antibiotics is a real global public health problem, especially in developing countries and Cameroon in particular [15]. *E. coli* is generally transmitted by the fecal-oral route, and MDR forms of *E. coli* ESBL are transmissible by contact with humans, animals, or the environment, or by ingestion of contaminated food or water, on the one hand, and overuse or inappropriate prescription of antibiotics on the other [16, 17].

The resistance profile, virulence factors, and ESBLs of enterovirulent *E. coli* strains in patients with enteric infections admitted to the oncology ward of the Laquintinie Hospital in Douala were determined in this study.

## 2. Materials and Methods

### 2.1. Biological Samples and Study Population

Participants in this study were cancer patients admitted to the oncology ward with enteric diseases, treated at the Laquintinie Hospital in Douala, which is a referral hospital in the city of Douala, Littoral Region of Cameroon. Fresh stool samples were collected from patients using sterile containers, and a total of 507 samples were collected between March 2021 and March 2023. These samples were immediately sent to the bacteriology laboratory after collection for culture. All raw data collected from the studies populations are available as Supporting Information S1–S4.

### 2.2. Stool Bacteriological Analysis

This analysis involved the isolation and identification of bacteria from stool samples. Each stool sample was diluted in sterile distilled water and cultured on EMB Levine agar (methylene blue eosin) (Liofilchem, Roseto, Italy) because of the characteristic appearance of *E. coli* on this medium and incubated at 37°C for 24 h. Based on morphological examination, colonies that appeared flat, very dark purple with generally a metallic sheen and lactose fermenting were preliminary flagged as *E. coli* and confirmed using biochemical tests using a suspension that inoculated the wells of the analytical profile index (API) 20E gallery (bioMérieux, Marcyl'Etoile, France) for the detection of twenty biochemical characters. The strains identified were tested for antibiotic susceptibility and preserved in glycerol broth at −80°C for molecular analysis of *E. coli* pathotypes (EAEC, EPEC, ETEC, EIEC, and STEC) and antibiotic resistance genes by polymerase chain reaction (PCR) [18].

### 2.3. Antimicrobial Susceptibility Testing

Antimicrobial susceptibility testing was performed on all isolates using the diffusion method as described by Kirby–Bauer on Mueller–Hinton agar (Titan Biotech LTD, Rajasthan, India), following the recommendations of the European Committee on antimicrobial susceptibility testing (EUCAST) [19, 20]. Antibiotic susceptibility testing of the isolates was performed according to the Kirby–Bauer method [14] and the antibiotics tested included imipenem (IMP) (10 μg), ceftazidime (CAZ) (30 μg), aztreonam (ATM) (30 μg), amoxicillin (AMX) (10 μg), amoxicillin/clavuranic acid (AMC) (20/10 μg), ceftriaxone (CTR) (30 μg), cefuroxin (CXM) (30 μg), gentamicin (GEN) (10 μg), amikacin (AMK) (30 μg), nitrofurantoin (NIT) (100 μg), ofloxacin (OFX) (5 μg), tetracycline (TET) (30 μg), colistin (COL) (30 μg), ciprofloxacin (10 μg), nalidixic acid (NAL) ( 10 μg), piperacillin (PRL) (10 μg), trimethoprim-sulfamethoxazole (COT) (10 μg), cefoxitin (FOX) (10 μg), cefotaxime (CTX) (10 μg), erythromycin (ERY) (10 μg), and fosfomycin (FOS) (10 μg). After incubation, the zones of inhibition around the antibiotic discs were measured and interpreted based on the stopping point criteria of the EUCAST [20]. Quality control of antibiotic discs (Singapore Bioscience PTE Ltd., Singapore) and incubation conditions were performed using *E. coli* ATCC 25922. Isolates showing resistance to three or more classes of antibiotics were multidrug-resistant bacteria [21].

### 2.4. DNA Extraction

DNA was extracted from fresh colonies using the heat shock technique. In 400 *μ*L of 1X Tris-ethylenediaminetetraacetic acid (EDTA) buffer, a loopful of fresh bacterial colonies was placed (0.1 M Tris-Cl and 0.01 M EDTA diluted 1:10). After 5 s of vortexing, the suspension was heated for 25 min at 95°C in a water bath. For 3 min, the heated suspension was centrifuged at 13,000 rpm. The supernatant containing the DNA was stored at −20°C for molecular analysis by PCR after being diluted tenfold [22].

### 2.5. Investigation of Virulence Genes by Simplex PCRs

The detection of STEC, EPEC, EAEC, ETEC, and EIEC was carried out using simplex PCR tests with specific primers (Table 1) for the identification of the following virulence markers: EPEC is based on the presence of the *bfpA* gene, *E. coli* are classified as ETEC based on the presence of *LT* genes, the *AggR* plasmid-encoded gene is used to classify EAEC *E. coli* isolates, and *E. coli* are classified as STEC and EIEC based on the presence of *Stx* and *IpaH* genes, respectively [6]. Five pathogenicity factors were tested using primers targeting four adhesins and one toxin (see Table 1). The adhesin genes tested were *bfpA, LT, IpaH,* and *AggR,* and the toxin gene was *Stx.* The required concentrations of the different components and the specific final volumes of the mixture were pipetted into the individual PCR tubes as required. In brief, 14.9 μL of deionized Sigma water (New England BioLabs, Ipswich, Massachusetts) was dispensed into each PCR tube, and the following was added: Master mix (2.5 μL buffer + 2 mM MgCl_2_ + 0.5 μL dNTP mix + 20 μM forward primer (1.0 μL) + 20 μM reverse primer (1.0 μL) + 0.1 μL standard taq DNA polymerase 5. 0 U) (New England Biolab, United Kingdom) was added to a 1.5 mL tube. These volumes were multiplied by the number of samples to be analyzed. 20 μL of the master mix was pipetted into each PCR tube, and 5.0 μL of DNA sample was then added to each PCR tube. Table 1 shows the primer sequences used and the PCR conditions as described in a previous study with slight modifications [6].

### 2.6. ESBL Gene PCR Screening

The PCR was run for the detection of the following ESBL genes: *bla*_*TEM*_, *bl*a_*Oxa*_, bl*a*_CTX‐*M*_, and *bla*_*SHV*_. The required concentrations of the different components and the specific final volumes of the mixture were pipetted into the individual PCR tubes as required. In brief, 14.9 μL of deionized Sigma water (New England BioLabs, Ipswich, Massachusetts) was dispensed into each PCR tube, and the following was added: Master Mix (2.5 μL buffer + 2 mM MgCl_2_ + 0.5 μL dNTP mix + 20 μM forward primer (1.0 μL) + 20 μM reverse primer (1.0 μL) + 0.1 μL standard taq DNA polymerase 5. 0 U) (New England Biolab, United Kingdom) was added to a 1.5 mL tube. These volumes were multiplied by the number of samples to be analyzed. 20 μL of the master mix was pipetted into each PCR tube and 5.0 μL of DNA sample was then added to each PCR tube in a Techne thermal cycler [22].  Table 2 shows the primer sequences used and PCR conditions as described by a previous study with slight modifications [23]. The amplified products were then subjected to electrophoresis in a 1.5% agarose gel and visualized using a transilluminator.

### 2.7. Ethics Approval

Ethical approvals were obtained from the National Committee for Ethics in Human Sciences Research (CNERSH), Yaoundé, Cameroon (No. 2022/09/129/CE/CNERSH/SP) and from the Institutional Ethics Committee of the University of Douala (CIE-UD) (Number: 3127/CEI-UDo/06/2022/T). Before data collection, the authorization from the director of Laquintinie Hospital in Douala was obtained. The information sheet outlining the purpose and details of the study was explained to each participant. Answers were also suggested to their questions about the study. Informed consent was obtained from all patients for the publication of all their data.

### 2.8. Statistical Analysis

This study provided results on antibiotic resistance in *E. coli* and the distribution of virulence genes and resistance genes in cancer and noncancer patients. Qualitative data were presented in the form of frequency distribution tables. The sensitivity profile was expressed as a percentage. The chi-square test and Fisher's exact test were used to compare *E. coli* resistance frequencies in cancer and noncancer patients. Risk estimates and Spearman correlation tests were applied to determine associations between virulence factors, resistance genes, and the AMR phenotype. A *p* value of < 0.05 was considered significant. The visual dashboard test was used to compare the 95% odds ratios of the ESBL gene in enterovirulent *E. coli* to infer a possible relationship between resistance genes, virulence factors, and the resistance phenotypic profile. All analyses were performed using Epi Info^TM^ version 7.2.4 software (CDC, 1600 Clifton Road, Atlanta, GA 303294027, United States of America).

## 3. Results

### 3.1. Prevalence of *E. coli* Bacterial Infections and Antibiotic Resistance Profile

A total of 202 isolates, representing a prevalence of 39.84% of *E. coli* infection, were obtained from the stools of the participants in this study. Of these isolates, 115 with a prevalence of 37.45% were from cancer patients, and 87 with a prevalence of 43.50% were from noncancer patients. The susceptibility of the *E. coli* isolates obtained from twenty-two different antibiotics was evaluated in this study (Table 3). It appears that the *E. coli* isolates showed significantly higher rates of resistance (*p* < 0.05) in cancer patients compared to noncancer patients to AMX (87.83% vs. 66.67%), AMC (59.13% vs. 27.59%), CXM (80.87% vs. 39.08%), PRL (95.65% vs. 51.72%), CTR (66,09% vs. 34.48%), CAZ (68.70% vs. 27.59%), CIP (46.09% vs. 31.03%), OFX (47.83% vs. 32.18%), NAL (60, 87% vs. 49.43%), COL (60.87% vs. 24.14%), TET (89.57% vs. 63.22%), and ERY (75.65% vs. 29.89%).

### 3.2. Frequency of Occurrence of Multidrug-Resistant (MDR) *E. coli* as a Function of Cancer Status

**Figure 1:**
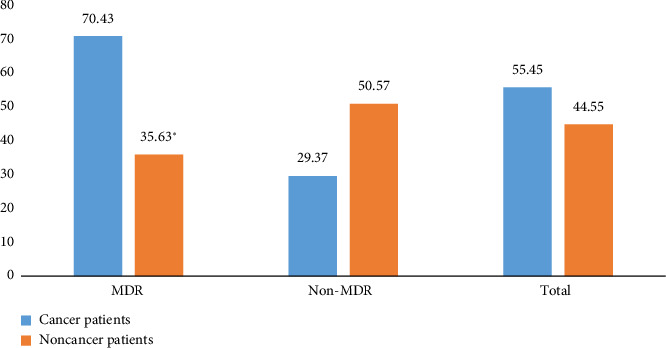
Frequency of multidrug-resistant (MDR) *Escherichia coli* isolated from cancer and noncancer patients. ^∗^*p* < 0.001.

The prevalence of MDR in *E. coli* in this study was 55.45%.  Figure 1 shows the frequency of the occurrence of MDR in different isolates from cancer and noncancer patients. *E. coli* isolates showed high MDR (70.43%) in cancer patients compared to noncancer patients (35.65%). *E. coli* isolates showed a significant increase in MDR phenotypes in cancer patients compared to noncancer patients (*p* < 0.05).

### 3.3. Association of Prior Exposure to Antibiotic Therapy in the Last Six Months With MDR of *E. coli*

Different risk factors for resistance in the study groups were presented in the study by Kengne et al. (2024) [24].  Table 4 shows the association between prior antibiotic exposure and MDR of *E. coli* in cancer and noncancer patients. *E. coli* MDRs were more likely to be observed in cancer patients who had been exposed to antibiotic therapy in the last six months than in cancer patients who had not been exposed (OR = 1.28; 95% CI: 0.53–3.05; *p*=0.364; Table 4), but no statistically significant association was found (*p* > 0.05).

### 3.4. Frequency of Enterovirulent *E. coli* According to the Clinical and Epidemiological Characteristics of Patients

**Figure 2:**
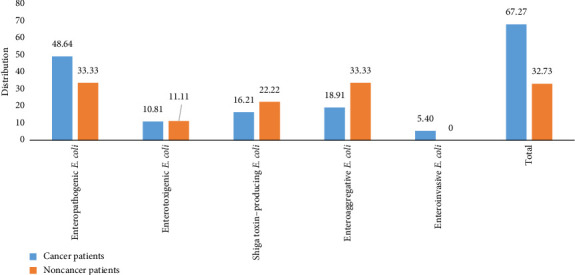
Distribution of enterovirulent *E. coli* according to cancer status.

The demographic and clinical characteristics of patients with enterovirulent *E. coli* are presented in Table 5. Based on the morphological characteristics of the colonies, which appeared flat, very dark purple with generally a metallic sheen and lactose fermentation, and biochemical tests using the API 20E gallery (bioMérieux, Marcyl'Etoile, France), a total of 202 *E. coli* isolates were obtained from 507 stool samples. Simplex PCR used to target virulent genes identified 55 enterovirulent *E. coli* isolates, of which 24 (43.63%) were EPEC, 13 (23.63%) were EAEC, 6 (10.90%) were ETEC, 10 (18.18%) were STEC, and 2 (3.63%) were EIEC. Of the 55 enterovirulent *E. coli*, 28 (50.91%) were detected in women compared with 27 (49.09%) in men. Enterovirulent *E. coli* was more prevalent in the 30–60 age group (61.82%), with a predominance of EPEC pathovar (*n* = 16; 66.67%). Diarrhea was observed in 78.18% (*n* = 43) of patients with enterovirulent *E. coli*, including 39.53% (*n* = 17) of patients infected with EPEC (Table 5). Molecular detection of enterovirulent *E. coli* according to cancer status (Figure 2) showed that EPEC was predominant in cancer patients (48.64% (*n* = 18) compared to 33.33% (*n* = 6) in noncancer patients, while ETEC, STEC, and EAEC were predominant in noncancer patients.

### 3.5. Distribution of Resistance Genes in Enterovirulent *E. coli* According to Cancer Status

Of the 55 isolates analyzed for ESBL production, 94.54% (52/55) harbored at least one ESBL gene. The most predominant gene in this study was the *bla*_TEM_ gene (80.00%, 43/55), followed by the *bla*_CTX−M_ gene (67.27%, 37/55) and the *bla*_OXA_ gene (24.63%, 24/55), and the least detected gene was the *bla*_SHV_ gene (36.36%, 20/55).  Table 6 shows the resistance genes harbored by enterovirulent *E. coli* in the different groups. The results showed that the *bla*_TEM_ gene was higher (*p* ≤ 0.001) in enterovirulent *E. coli* isolated from cancer patients (91.89%, *n* = 34) than in those isolated from noncancer patients (55.55%, *n* = 10). The results also showed that the *bla*_CTX−M_ gene was significantly higher (*p* ≤ 0.001) in enterovirulent *E. coli* isolated from cancer patients (87.78%, *n* = 31) than in enterovirulent *E. coli* isolated from noncancer patients (33.33%, *n* = 6).

### 3.6. Association Between Virulence Genes and Beta-Lactamase Genes in Enterovirulent *E. coli*

The association between virulence factors and resistance genes in *E. coli* isolates is presented in Table 7. No statistically significant correlation was found between the presence of virulence factors and ESBL gene carriage (*p* > 0.05). Nevertheless, EPEC (carrying the bfpA factor) had a 2.05-fold risk of carrying the *bla*_CTX−M_ genes.

### 3.7. Association Between Virulence Genes and the Phenotypic Resistance Profile of Enterovirulent *E. coli*

The correlation between carriage of the virulence genes *bfpA, LT, AggR,* and *Stx* and antibiotic resistance in *E. coli* isolates was assessed and presented in Table 8. Table 8 shows that there was a significant correlation between carriage of the *bfpA* gene and antibiotic resistance to CTR (OR = 3.85; 95% CI: 0.936–15.862; *p*=0.049; Table 8) and a significant association between the presence of the *AggR* gene and antibiotic resistance to CTR (OR = 0.27; 95% CI: 0.072–1.043; *p*=0.035; Table 8). A significant correlation was also observed between carriage of the *bfpA* gene and antibiotic resistance to NIT (OR = 3.52; 95% CI: 1.062–11.699; *p*=0.034).

### 3.8. Association Between Resistance Genes and the Phenotypic Resistance Profile of Enterovirulent *E. coli*

The correlation between the resistance genes *bla*_TEM_, *bla*_OXA_, *bla*_CTX−M_, and *bla*_SHV_ and antibiotic resistance in enterovirulent *E. coli* isolates is presented in Table 9. Table 9 shows that significant correlations were found between the *bla*_TEM_ gene and AMC (OR = 4.66; 95% CI: 1.155–18.855; *p*=0.029), CTX (OR = 4.40; 95% CI: 1.048–18.511; *p*=0.058), and CXM (OR = 8.33; 95% CI: 1.733–40.051; *p*= 0.010). Second, there was a positive association between the *bla*_CTX−M_ gene and resistance to antibiotics such as CXM (OR = 5.66; 95% CI: 1.221–26.281; *p*=0.026) and CAZ (OR = 3.62; 95% CI: 1.080–12.167; *p*=0.035). Finally, there was a significant correlation between the *bla*_OXA_ gene and CAZ resistance (OR = 3.61; 95% CI: 0.995–13.104; *p*=0.041; Table 9).

The evaluation of the correlation between carriage of the *bla*_TEM_ + *bla*_CTX−M_, bla_TEM_ + *bla*_OXA_, *bla*_TEM_ + *bla*_SHV_, *bla*_CTX−M_ + *bla*_OXA_, and *bla*_CTX−M_ + *bla*_SHV_ resistance genes and antibiotic resistance in enterovirulent *E. coli* isolates is presented in Table 10. Simultaneous carriage of two *bla*_CTX−M_ + *bla*_SHV_ resistance genes showed a positive association with CAZ resistance (OR = 8.32; 95% CI: 0.990–69.912; *p*=0.023). Table 10 also shows that the presence of both *bla*_TEM_ + *bla*_CTX−M_ genes correlated with CXM resistance (OR = 5.97; 95% CI: 1.110–32.089; *p*=0.029). Carriage of the *bla*_TEM_ + *bla*_SHV_ resistance genes was also correlated with CXM resistance (OR = 4.26; 95% CI: 0.489–37.205; *P* = 0.041). A statistically significant correlation was obtained between *bla*_TEM_ + *bla*_OXA_ and CXM resistance (OR = 6.72; 95% CI: 0.776–58.172; *p*=0.030). A risk correlation was also observed between the two *bla*_CTX−M_ + *bla*_SHV_ genes and AMX (OR = 2.22) and CTX (OR = 4.76).

### 3.9. MDR Profile of Enterovirulent *E. coli*

**Figure 3:**
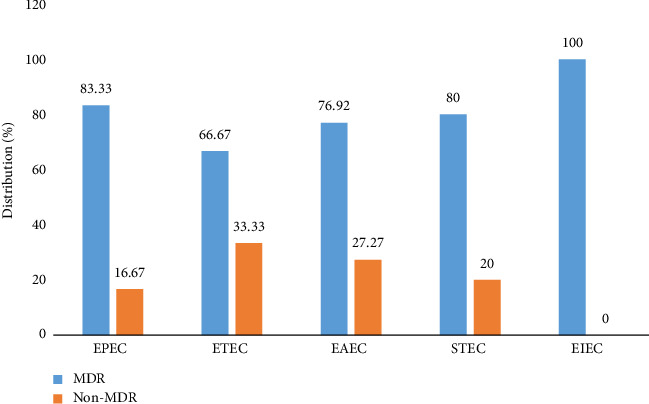
Characteristics of enterovirulent *E. coli* according to their multidrug resistance status. MDR: multidrug resistance, STEC: Shiga toxin–producing *E. coli*, EPEC: enteropathogenic *E. coli*, EAEC: enteroaggregative *E. coli*, ETEC: enterotoxigenic *E. coli*, DAEC: diffuse adherent *E. coli*, and EIEC: enteroinvasive *E. coli*.

The characteristics of enterovirulent *E. coli* according to MDR status show that all enterovirulent *E. coli* were more likely to be MDR than non-MDR (Figure 3).

## 4. Discussion

*E. coli* is a bacterium capable of causing intestinal infections ranging from mild to life-threatening. The pathogenicity of this bacterium depends on many factors, including virulence properties and AMR [25, 26]. Enterovirulent *E. coli* is not generally studied in Cameroon due to the lack of suitable technical facilities, such as molecular diagnostic techniques. Consequently, the treatment of enterovirulent *E. coli* infections in patients in the Littoral region, Douala, Cameroon in general, and cancer patients in particular remains a serious public health issue. Furthermore, there is a lack of rigorous epidemiological evidence demonstrating the coexistence of resistance and virulence in these patients. Stool samples were collected in this study from cancer and noncancer patients suffering from enteric diseases and managed at the Laquintinie Hospital in Douala, Littoral Region of Cameroon. This study is one of the first based on the search for enterovirulent *E. coli* in the population of the city of Douala.

This study showed a 39.84% prevalence of *E. coli* infection obtained from the stools of participants, with a 37.45% prevalence in cancer patients and a 43.50% prevalence in noncancer patients. It determined the susceptibility profile of *E. coli* isolates from fecal samples of cancer and noncancer patients at the Laquintinie Hospital in Douala to provide up-to-date information on local resistance data and antimicrobials for this pathogen. A low rate of resistance to IMP was observed in this study, which may be because it is a very potent drug used only in hospitals and not as a first-line treatment in outpatient clinics. Several studies have also reported low rates of resistance to imipenem, such as Sedighi et al. [27] in Iran, Muktakhaparkuntikar [28] in Bulgaria, and Gad [29] in Egypt. It appears that *E. coli* isolates showed significantly (*p* < 0.05) higher resistance rates in cancer patients compared to noncancer patients to AMX (87.83% versus. 66.67%), AMC (59.13% versus 27.59%), PRL (95.65% versus 51.72%), CIP (46.09% versus 31.03%), OFX (47.83% versus 32.18%), COL (60.87% versus 24.14%), and TET (89.57% versus 63.22%). These results show a marked resistance of *E. coli* isolates in cancer patients compared with noncancer patients and highlight the very high resistance of *E. coli* to beta-lactam antibiotics. This observation corroborates that of Mahmoud et al. who also obtained high levels of resistance to beta-lactam antibiotics in their study in Egypt [30]. This is thought to be due to the empirical antibiotic-based treatment commonly used in oncology departments, prolonged hospital stays by cancer patients, and the various resistance mechanisms developed by *E. coli*, such as enzymatic inactivation through the production of beta-lactamases, target modification, and efflux [31].

The molecular analysis enabled us to identify 55 enterovirulent *E. coli* isolates, of which 43.63% were EPEC, 23.63% were EAEC, 10.90% were ETEC, 18.18% were STEC, and 3.63% were EIEC, with a predominance of EPEC in cancer patients. These results are like those obtained in Mbouda, Cameroon, by Marbou et al. which showed that EPEC was the most prevalent, followed by EAEC and STEC [10, 32], unlike those obtained in Moyo, Tanzania [33]. Diarrhea was observed in 78.18% of patients with enterovirulent *E. coli*, including 39.53% of patients infected with EPEC. The incidence of EPEC was higher in patients presenting with symptoms of abdominal pain, nausea, fever, and diarrhea. In our study, EPEC (43.63%; *n* = 24) was found to be the most abundant pathotype in gastrointestinal disorders in Douala. It has been reported that ETEC produces a heat-labile toxin (LT) whose sequence, antigenicity, and function are like the cholera toxin responsible for food poisoning in humans [34]. Certain strains of EPEC can colonize the small intestine and adhere to the mucosa, causing obliteration of the microvilli, as an EPEC adhesion factor (EAF) encoding a plasmid plays a key role in the pathogenesis of EPEC [35]. The frequency of *E. coli* pathotypes and virulent genes depends on geographical location. In Tehran, Shahrokhi et al. showed that ST was the most common toxin type, and this was also documented in several countries [36, 37].

The production of ESBLs is an important resistance mechanism that inhibits antimicrobial actions against infections caused by Enterobacteriaceae. The prevalence of ESBL-producing bacteria varies from country to country, and in Cameroon, ESBLs are considered a serious threat to currently available antimicrobial agents. The presence of ESBL-producing bacteria was reported in this study. Molecular analysis revealed that the most predominant gene in this study was the *bla*_TEM_ gene (80.00%, 43/55), followed by the *bla*_CTX−M_ gene (67.27%, 37/55) of the resistant strains. This result could be due to the increased frequency of CTX and CAZ consumption, which could contribute to the emergence of genes coding for CTX-M enzymes among *E. coli* strains in hospitals. The reason for the increase in the appearance of ESBL-producing organisms is probably due to the overuse of antibiotics [38]. On the other hand, studies have shown similar results, as Azargun et al. [39] in Iran reported that the TEM gene was the major ESBL gene in *E. coli* isolates, followed by the CTX-M gene and the SHV gene, where the rates for TEM, CTX-M, and SHV were 75.6%, 78.6%, and 33.3%, respectively. The *bla*_OXA_ gene (24.63%, 24/55) and the *bla*_SHV_ gene (36.36%, 20/55) were less frequent in our study. In the present study, *bla*_TEM_ was the most common gene detected in ESBL-producing *E. coli.* This is consistent with a study conducted in Congo, Brazzaville, in which the TEM-type gene was the main beta-lactamase type and CTX-M was the second [40, 41]. In Burkina Faso, the most common ESBL resistance gene was *bla*_TEM_ (26.2%) followed by *bla*_SHV_ (5.9%) in Enterobacteriaceae [42].

In this study, numerous associations between virulent factors and AMR were identified. A significant correlation was found between carriage of the *bfpA* gene and antibiotic resistance to CTR (OR = 3.85; 95% CI: 0.936–15.862; *p*=0.049) and a significant association between the presence of the *AggR* gene and antibiotic resistance to CTR (OR = 0.27; 95% CI: 0.072–1.043; *p*=0.035). A significant correlation was also observed between *bfpA* gene carriage and antibiotic resistance to NIT (OR = 3.52; 95% CI: 1.062–11.699; *p*=0.034). No association was observed between virulent genes and antimicrobial-resistant genes. Correlations between the resistance genes *bla*_TEM_, *bla*_OXA_, *bla*_CTX−M_, and *Bla*_*S*HV_ and antibiotic resistance in enterovirulent *E. coli* isolates were observed in this study. These associations may be explained by the increase in antibiotic consumption, which could consequently contribute to the emergence of genes encoding the TEM, OXA, CTX-M, and SHV enzymes among *E. coli* strains [43, 44]. There are three main reasons for these correlations: the coexpression of virulence factors and antibiotic resistance; the effect of a powerful immune system, which leads to increased expression of virulence factors; and the transfer of genes onto plasmids [45]. It appears that the expression of virulence genes is linked to antibiotic resistance, so that any increase in the expression of virulence genes in isolates will lead to an increase in their resistance to antibiotics.

The results of this study are of great importance in terms of public health, as bacterial infections including those caused by *E. coli* and their drug resistance still constitute a serious health issue globally [46–52]. The study focused on the prevalence of fecal carriage of ESBL-producing enterovirulent *E. coli* with a high rate of MDR in cancer patients. Although it presents data on cancer patients with limited information on the resistance of enterovirulent *E. coli* to commonly used antibiotics and on the faecal carriage of ESBLs, certain limitations need to be recognized. No other molecular studies, such as sequencing of the ESBLs studied in this work, have been carried out. Sequencing of the ESBLs studied in this work would be important in order to confirm TEM, SHV, or OXA ESBLs from other variants that are simple penicillinases. Future studies will therefore be important to determine the risks associated with *E. coli* carrying antibiotic resistance genes and the ability to transfer these genes to other bacteria, as well as the correlation between antibiotic resistance and the phylogenetic groups of *E. coli* and sequencing to determine whether they are ESBLs or penicillinases.

## 5. Conclusion

This study is one of the first to reveal the real existence of fecal carriage of ESBL-producing enterovirulent *E. coli* isolates from cancer patients in the city of Douala, with a high rate of MDR in the latter. The presence of EPEC, EAEC, ETEC, STEC, and EIEC was observed. A high antibiotic resistance phenotype was observed in cancer patients, with resistance to commonly used antibiotics, including AMX, AMC, PRL, CIP, COL, and TET. There is a high prevalence of 94.54% of *E. coli* ESBL isolates, with a predominance of the *bla*_TEM_ gene (80.00%). In addition, the coexistence of two different ESBL genes was frequently detected in certain bacterial pathogens. Several associations between virulence factors and the AMR phenotype, as well as between resistance genes and the AMR phenotype, have been observed. Monitoring of antibiotic resistance in bacterial strains should be continuous and systematic to define therapeutic strategies adapted to the epidemiological data given in cancer patients.

## Figures and Tables

**Table 1:** List of primers used for *E. coli* virulence typing by PCR.

Target genes	Primers	Nucleotide sequences (5′-3′)	Amplicon size	PCR conditions (40 cycles)	Reference
*Adhesin genes*					
*bfpA*/EPEC	*bfPA*-F	5′-AATGGTGCTTGCGCTTGCTGC-3′	326 bp	2 min at 94°C, 30 s at 92°C, 30 s at 59°C, 5 min at 72°C, 30 s at 72°C	[6]
	*bfPA*-R	5′-GCCGCTTTATCCAACCTGGTA-3′			
*LT/ETEC*	*LT*-F	5′-GCACACGGAGCTCCTCAGTC-3′	218pb	2 min at 94°C, 30 s at 92°C, 30 s at 59°C, 5 min at 72°C, 30 s at 72°C	
	*LT*-R	5′-TCCTTCATCCTTTCAATGGCTTT-3′			
*AggR/EAEC*	*AggR*-F	5′-GTATACACAAAAGAAGGAAGC-3′	254pb	2 min at 94°C, 30 s at 92°C, 30 s at 59°C, 5 min at 72°C, 30 s at 72°C	
	*AggR*-R	5′-ACAGAATCGTCAGCATCAGC-3′			
*IpaH/EIEC*	*IpaH*-F	5′-CTCGGCACGTTTTAATAGTCTGG-3′	933 bp	5 min at 94°C, 30 s at 92°C, 30 s at 62°C, 5 min at 72°C, 30 s at 72°C	
	*IpaH*-R	5′-GTGGAGAGCTGAAGTTTCTCTGC-3′			

*Toxin genes*					
*Stx/STEC*	*VT com*-F	5′-GAGCGAAATAATTTATATGTG-3′	518 bp	7 min at 94°C, 30 s at 92°C, 30 s at 60°C, 5 min at 72°C, 30 s at 72°C	[6]
	*VT com*-R	5′-TGATGATGGCAATTCAGTAT-3′			

Abbreviations: DAEC, diffuse adherent *E. coli*; EAEC, enteroaggregative *E. coli*; EIEC, enteroinvasive *E. coli*; EPEC, enteropathogenic *E. coli*; ETEC, enterotoxigenic *E. coli*; STEC, Shiga toxin–producing *E. coli*.

**Table 2:** Specific PCR primers used in this study for the determination of ESBL genes.

ESBL gene	Primers	Sequence (5′-3′)	Amplicon size	PCR conditions (35 cycles)	Reference
*bla*_TEM_	*bla*_TEM−F_	5′CGCCGCATACACTATTCTCAGAATGA3′ 5′ACGCTCACCGGCTCCAGATTTAT-3′	445 bp	7 min at 94°C, 30 s at 94°C, 30 s at 58°C, 10 min at 68°C, 1 min at 68°C	[23]
	*bla*_TEM−R_				
*bla*_OXA_	*bla*_OXA−F_	5′ACACAATACATATCAACTTCGC3′	813pb	7 min at 94°C, 30 s at 94°C, 30 s at 58°C, 10 min at 68°C, 1 min at 68°C	
	*bla*_OXA−R_	5′-AGTGTGTTTAGA ATGGTGATC-3′			
*bla*_CTX−M_	*bla*_CTX−M−F_	5′-ATGTGCAGYACCAGTAARGTKATGGC3′	593pb	7 min at 94°C, 30 s at 94°C, 30 s at 55°C, 10 min at 68°C, 1 min at 68°C	
	*bla*_CTX−M−R_	5′-TGGGTRAARTARGTSACCAGAAYCAGCGG-3′			
*bla*_SHV_	*bla*_SHV−F_	5′-CTTTATCGGCCCTCA CTCAA-3′	237 bp	7 min at 94°C, 30 s at 94°C, 30 s at 62°C, 10 min at 68°C, 1 min at 68°C	
	*bla*_SHV−R_	5′-AGGTGCTCATCATGGGAAAG-3′			

**Table 3:** Resistance profile of *E. coli* according to cancer status.

Antibiotics		*E. coli n *= 202 (%)			
		Cancer patients *n* = 115 (%)	Noncancer patients *n* = 87 (%)	*X* ^2^	*p* value
IMP	S	112 (97.39)	85 (97.70)	13.20	< 0.001
	R	3 (2.61)	2 (2.30)		

AMX	S	14 (12.17)	29 (33.33)	12	< 0.001
	R	101 (87.83)	58 (66.67)		

AMC	S	42 (36.52)	63 (72.41)	26.87	< 0.001
	I	5 (4.35)	0 (0.00)		
	R	68 (59.13)	24 (27.59)		

FOX	S	42 (36.52)	55 (63.22)	13.09	< 0.001
	R	73 (63.48)	32 (36.78)		

CTX	S	27 (23.48)	51 (58.62)	24.34	< 0.001
	R	88 (76.52)	36 (41.38)		

CXM	S	19 (16.52)	52 (59.77)	40.64	< 0.001
	I	3 (2.61)	1 (1.15)		
	R	93 (80.87)	34 (39.08)		

PRL	S	5 (4.35)	41 (47.13)	53.58	< 0.001
	I	0 (0.00)	1 (1.15)		
	R	110 (95.65)	45 (51.72)		

CTR	S	38 (33.04)	57 (65.52)	21.29	< 0.001
	I	1 (0.87)	0 (0.00)		
	R	76 (66.09)	30 (34.48)		

CAZ	S	31 (26.96)	63 (72.41)	42.19	< 0.001
	I	5 (4.35)	0 (0.00)		
	R	79 (68.70)	24 (27.59)		

AMK	S	105 (91.30)	84 (96.55)	2.50	0.280
	I	1 (0.87)	0 (0.00)		
	R	9 (7.83)	3 (3.45)		

GEN	S	109 (94.78)	85 (97.70)	0.50	0.781
	R	6 (5.22)	2 (2.30)		

CIP	S	58 (50.43)	60 (68.97)	8.77	0.01
	I	4 (3.48)	0 (0.00)		
	R	53 (46.09)	27 (31.03)		

OFX	S	55 (47.83)	59 (67.82)	10.23	0.006
	I	5 (4.35)	0 (0.00)		
	R	55 (47.83)	28 (32.18)		

NAL	S	45 (39.13)	44 (50.57)	2.18	0.06
	R	70 (60.87)	43 (49.43)		

COT	S	84 (73.04)	62 (71.26)	0.10	0.90
	I	1 (0.87)	1 (1.15)		
	R	30 (26.09)	24 (27.59)		

COL	S	43 (37.39)	64 (73.56)	27.14	< 0.001
	I	2 (1.74)	2 (2.30)		
	R	70 (60.87)	21 (24.14)		

TET	S	12 (10.43)	32 (36.78)	18.66	< 0.001
	R	103 (89.57)	55 (63.22)		

NIT	S	83 (72.17)	72 (82.76)	3.43	0.170
	I	4 (3.48)	1 (1.15)		
	R	28 (24.35)	14 (16.09)		

ATM	S	62 (53.91)	68 (78.16)	16.77	< 0.001
	I	1 (0.87)	3 (3.45)		
	R	52 (45.22)	16 (18.39)		

FOS	S	64 (55.65)	85 (97.70)	45.25	< 0.001
	I	1 (0.87)	0 (0.00)		
	R	50 (43.48)	2 (2.30)		

ERY	S	25 (21.74)	59 (67.82)	43.35	< 0.001
	I	3 (2.61)	2 (2.30)		
	R	87 (75.65)	26 (29.89)		

Abbreviations: AMC, amoxicillin/clavuranic acid; AMK, amikacin; AMX, amoxicillin; ATM, aztreonam; CAZ, ceftazidime; CIP, ciprofloxacin; COL, colistin; COT, trimethoprim-sulfamethoxazole; CTR, ceftriaxone; CTX, cefotaxime; CXM, cefuroxin; ERY, erythromycin; FOS, fosfomycin; FOX; cefoxitin; GEN, gentamicin; I, intermediate; IPM, imipenem; NAL, nalidixic acid; NIT, nitrofurantoin; OFX, ofloxacin; PRL, piperacillin; R, resistant; S, sensitive; TET, tetracycline.

**Table 4:** Association between prior antibiotic exposure and multidrug resistance in *Escherichia coli*.

		MDR *E. coli n* = 113 (%)	Non-MDR *E. coli n* = 89 (%)	OR (95% CI)	*p* value
Cancer patients	Previous exposure to antibiotic therapy	59 (52.21)	22 (24.71)	1.28 (0.53–3.05)	0.364
	No previous exposure to antibiotic therapy	23 (20.35)	11 (12.35)		

Noncancer patients	Previous exposure to antibiotic therapy	8 (7.07)	16 (17.97)	0.86 (0.38–2.34)	0.494
	No previous exposure to antibiotic therapy	23 (20.35)	40 (44.94)		

Abbreviations: CI, confidence interval; *E. coli*, *Escherichia coli*; MDR, multidrug resistance; OR, odds ratio.

**Table 5:** Clinical and epidemiological characteristics of patients with enterovirulent *E. coli* identified in their stools.

Features	*Enterovirulent E. coli n* = 55 (%)	EPEC *n* = 24 (%)	ETEC *n* = 6 (%)	EAEC *n* = 13 (%)	STEC *n* = 10 (%)	EIEC *n* = 2 (%)
*Sex*						
Male	27 (49.09)	12 (50.0)	4 (66.67)	4 (30.77)	6 (60.0)	1 (50.0)
Female	28 (50.91)	12 (50.0)	2 (33.33)	9 (69.23)	4 (40.0)	1 (50.0)

*Age range*						
< 30 years	6 (10.91)	2 (8.33)	1 (16.67)	3 (23.08)	0 (0.0)	0 (0.0)
30–60 years	34 (61.82)	16 (66.67)	3 (50.0)	7 (53.85)	7 (70.0)	1 (50.0)
> 60 years	15 (27.27)	6 (25.00)	2 (33.33)	3 (23.08)	3 (30.0)	1 (50.0)

*Symptoms*						
Fever	27 (49.09)	10 (37.04)	3 (11.11)	7 (25.93)	6 (22.22)	1 (3.50)
Nausea	23 (41.82)	10 (43.48)	2 (8.70)	6 (25.09)	5 (21.74)	0 (0.0)
Vomiting	12 (21.82)	4 (33.33)	0 (0.0)	6 (50.0)	2 (16.67)	0 (0.0)
Abdominal pain	40 (72.73)	14 (35.00)	4 (10.0)	11 (27.50)	9 (22.50)	2 (5.00)
Diarrhea	43 (78.18)	17 (39.53)	3 (6.98)	12 (27.91)	10 (23.26)	1 (2.33)

Abbreviations: DAEC, diffuse adherent *E. coli*; EAEC, enteroaggregative *E. coli*; EIEC, enteroinvasive *E. coli*; EPEC, enteropathogenic *E. coli*; ETEC, enterotoxigenic *E. coli*; STEC, Shiga toxin–producing *E. coli*.

**Table 6:** Frequency of ESBL genes among *E. coli enterovirulent* isolates based on cancer status.

ESBL genes	Frequency of ESBL genes	Cancer patients *N* = 37 (%)	Noncancer patients *N* = 18 (%)	*x* ^2^	*P* value
*Single ESBL gene*					
*bla*_TEM_	44 (80.00)	34 (91.89)	10 (55.55)	11.49	< 0.001
*bla*_OXA_	24 (43.63)	20 (54.05)	4 (22.22)	5.58	0.010
*bla*_CTX−M_	37 (67.27)	31 (87.78)	6 (33.33)	15.26	< 0.001
*bla*_SHV_	20 (36.36)	18 (48.64)	2 (11.11)	7.94	0.002

*Two or more ESBL genes*					
*bla*_TEM_ *+bla*_CTX–M_	31 (56.36)	29 (78.37)	2 (11.11)	23.38	< 0.001
*bla*_TEM_ *+bla*_OXA_	22 (40.00)	19 (51.35)	3 (16.66)	6.65	0.005
*bla*_TEM_ *+bla*_SHV_	17 (30.90)	16 (43.24)	1 (5.55)	8.56	< 0.001
*bla*_CTX−M_ *+bla*_OXA_	20 (36.36)	18 (48.64)	2 (11.11)	7.94	0.002
*bla*_CTX−M_ *+bla*_SHV_	14 (25.45)	14 (37.83)	0 (0.00)	9.58	< 0.001

**Table 7:** Correlation between virulence factors and ESBL genes.

Markers	*BfpA/EPEC*		*LT/ETEC*		*AggR/EAEC*		*Stx/STEC*	
	Odds ratio (LI–UI)	*p* value	Odds ratio (LI–UI)	*p* value	Odds ratio (LI–UI)	*p*-value	Odds ratio (LI–UI)	*p* value
*bla*_OXA_	0.91 (0.31–2.67	0.547	0.59 (0.09–3.53)	0.448	1.09 (0.31–3.83)	0.444	1.31 (0.33–5.20)	0.481
*bla*_CTX−M_	2.05 (0.64–6.56)	0.174	2.5 (0.26–23.23)	0.233	0.66 (0.17–2.43)	0.274	0.37 (0.09–1.52)	0.153
*bla*_TEM_	0.69 (0.19–2.49)	0.404	1.15 (0.11–11.12)	0.693	0.38 (0.08–1.66)	0.115	NA	0.103
*bla*_SHV_	0.83 (0.27–2.52)	0.485	0.83 (0.13–5.01)	0.608	1.08 (0.13–5.01)	0.448	0.68 (0.15–2.99)	0.449

*Note:* The *p* value is given at 95% CI and significant at ≤ 0.05.

Abbreviations: DAEC, diffuse adherent *E. coli*l; EAEC, enteroaggregative *E. coli*; EIEC, enteroinvasive *E. coli*; EPEC, enteropathogenic *E. coli*; ETEC, enterotoxigenic *E. coli*; LI, lower interval; NA, not applicable; STEC, Shiga toxin–producing *E. coli*; UI, upper interval.

**Table 8:** Association between virulence factors and the genotypic resistance profile of enterovirulent *E. coli*.

Antibiotic-resistant	*bfpA/EPEC*		*LT/ETEC*			*AggR/EAEC*			*Stx/STEC*	
Patterns	Odds ratio (LI–UI)	*p* value	Odds ratio (LI–UI)		*p*-value	Odds ratio (LI–UI)		*p* value	Odds ratio (LI–UI)	*p* value
AMX	0.53 (0.107–2.661)	0.355	ND		0.423	0.35 (0.064–1.829)		0.204	ND	0.223
AMC	2.06 (0.674–6.962)	0.152	0.22 (0.036–1.338)		0.099	0.80 (0.220–2.901)		0.490	1.28 (0.291–5.677)	0.523
FOX	1.55 (0.443–5.446)	0.354	0.64 (0.105–3.995)		0.482	1.66 (0.394–7.041)		0.369	3.65 (0.419–31.839)	0.206
CTX	0.72 (0.199–2.598)	0.428	1.44 (0.152–13.725)		0.608	0.52 (0.129–2.162)		0.295	2.91 (0.330–25.630)	0.296
CXM	0.96 (0.228–4.051)	0.618	0.97 (0.100–9.507)		0.676	0.30 (0.067–1.366)		0.121	NA	0.139
PRL	3.40 (0.355–32.680)	0.265	0.44 (0.041–4.793)		0.451	0.16 (0.024–1.135)		0.071	NA	0.351
CTR	3.85 (0.936–15.862)	**0.049**	0.289 (0.051–1.641)		0.165	0.27 (0.072–1.043)		**0.035**	1.45 (0.269–7.839)	0.503
CAZ	0.81 (0.259–2.583)	0.478	0.88 (0.145–5.353)		0.610	1.66 (0.394–7.041)		0.369	0.60 (0.147–2.520)	0.368
CIP	1.49 (0.509–4.371)	0.323	0.40 (0.681–2.436)		0.283	1.06 (0.304–3.691)		0.589	0.87 (0.222–3.446)	0.561
OFX	1.37 (0.462–4.073)	0.384	0.31 (0.052–1.900)	0.192		1.20 (0.335–4.288)	0.520		1.09 (0.271–4.430)	0.593
NAL	1.42 (0.433–4.705)	0.388	0.36 (0.064–2.019)	0.229		0.90 (0.232–3.488)	0.567		0.947 (0.211–4.241)	0.609
COT	0.69 (0.228–2.098)	0.356	0.29 (0.031–2.673)	0.247		0.64 (0.184–2.243)	0.352		1.81 (0.455–7.217)	0.307
TET	1.17 (0.180–7.680)	0.622	0.44 (0.141–4.793)	0.4331		0.42 (0.062–2.857)	0.337		NA	0.351
NIT	3.52 (1.062–11.699)	**0.034**	NA	0.095		0.60 (0.142–2.534)	0.369		0.50 (0.094–2.653)	0.338
ERY	1.45 (0.372–5.706)	0.423	0.45 (0.071–2.847)	0.344		1.50 (0.280–8.026)	0.486		1.00 (0.180–5.545)	0.648

*Note:* The *p* value is given at 95% CI and significant at ≤ 0.05. In bold: positive association between antibiotic and the virulent gene.

Abbreviations: AMC, amoxicillin/clavuranic acid; AMX, amoxicillin; CAZ, ceftazidime; CIP, ciprofloxacin, COT, trimethoprim-sulfamethoxazole; CTR, ceftriaxone; CTX, cefotaxime; CXM, cefuroxin; EAEC, enteroaggregative *E. coli*; EPEC, enteropathogenic *E. coli*; ERY, erythromycin; ETEC, enterotoxigenic *E. coli*; FOX, cefoxitin; LI, lower interval; NA, not applicable; NAL, nalidixic acid; NIT, nitrofurantoin; OFX, ofloxacin; PRL, piperacillin; STEC, Shiga toxin–producing *E. coli*; TET, tetracycline; UI, upper interval.

**Table 9:** Correlation between ESBL genes and the resistance profile of enterovirulent *E. coli* to *β*-lactam antibiotics.

Antibiotic-resistant	*bla*_TEM_		*bla*_CTX−M_		*bla*_OXA_		*bla*_SHV_	
Patterns	Odds ratio (LI–UI)	*p* value	Odds ratio (LI–UI)	*p* value	Odds ratio (LI–UI)	*p* value	Odds ratio (LI–UI)	*p* value
AMC	4.66 (1.155–18.855)	**0.029**	1.89 (0.588–6.072)	0.218	2.16 (0.674–6.962)	0.152	0.96 (0.305–3.072)	0.591
AMX	3.75 (0.701–20.080)	0.078	3.23 (0.644–16.382)	0.148	5.52 (0.616–49.395)	0.100	1.50 (0.263–8.553)	0.497
FOX	1.94 (0.471–8.009)	0.285	2.72 (0.777–9.566)	0.104	1.55 (0.443–5.446)	0.354	2.59 (0.627–10.742)	0.152
CTX	4.40 (1.048–18.511)	**0.048**	2.58 (0.694–9.605)	0.137	1.73 (0.454–6.652)	0.316	1.96 (0.463–8.300)	0.283
CXM	8.33 (1.733–40.051)	**0.010**	5.66 (1.221–26.281)	**0.026**	3.20 (0.601–17.121)	0.147	2.25 (0.419–12.06)	0.286
PRL	7.87 (1.128–57.933)	**0.049**	NA	**0.002**	NA	**0.048**	0.84 (0.128–5.530)	0.605
CTR	3.24 (0.803–13.072)	0.097	1.19 (0.334–4.286)	0.513	0.70 (0.209–2.394)	0.401	0.69 (0.200–2.388)	0.391
CAZ	2.22 (0.570–8.655)	0.208	3.62 (1.080–12.167)	**0.035**	3.61 (0.995–13.104)	**0.041**	2.36 (0.649–8.608)	0.153

*Note:* The *p* value is given at 95% CI and significant at ≤ 0.05. In bold: positive association between antibiotic and the resistance gene.

Abbreviations: AMC, amoxicillin/clavuranic acid; AMX, amoxicillin; CAZ, ceftazidime; CTR, ceftriaxone, CTX, cefotaxime; CXM, cefuroxin; FOX, cefoxitin; LI, lower interval; NA, not applicable; PRL, piperacillin; UI, upper interval.

**Table 10:** Correlation between two ESBL genes and the *β*-lactam resistance profile of enterovirulent *E. coli*.

Antibiotic-resistant	bla_CTX−M_ + bla_OXA_		bla_CTX−M_ + bla_SHV_		bla_TEM_ + bla_CTX−M_		bla_TEM_ +bla_OXA_		bla_TEM_+bla_SHV_	
Patterns	Odds ratio (LI–UI)	*P* value	Odds ratio (LI–UI)	*P* value	Odds ratio (LI–UI	*P* value	Odds ratio (LI–UI)	*P* value	Odds ratio (LI–UI)	*P* value
AMC	1.37 (0.425–4.472)	0.408	0.93 (0.262–3.322)	0.579	2.43 (0.780–7.581)	0.103	2.50 (0.745–8.424)	0.111	1.40 (0.407–4.809)	0.414
AMX	3.93 (0.425–35.288)	0.193	2.22 (0.244–20.329)	0.420	2.14 (0.583–7.859)	0.202	4.66 (0.521–41.802)	0.141	3.00 (0.332–27.087)	0.293
FOX	1.05 (0.293–3.677)	0.608	2.48 (0.481–12.814)	0.229	2.08 (0.608–7.131)	0.192	1.95 (0.526–7.275)	0.245	1.90 (0.454–7.949)	0.295
CTX	1.18 (0.307–4.572)	0.543	4.76 (0.556–40.844)	0.068	2.14 (0.583–7.859)	0.202	2.37 (0.563–10.010)	0.194	2.67 (0.518–13.845)	0.199
CXM	2.25 (0.419–12.064)	0.286	NA	0.055	5.97 (1.110–32.089)	**0.029**	6.72 (0.776–58.172)	**0.030**	4.26 (0.489–37.205)	**0.041**
CTR	0.46 (0.134–1.599)	0.181	0.50 (0.135–1.894)	0.248	1.41 (0.417–4.772)	0.401	1.27 (0.362–4.483)	0.479	0.48 (0.138–1.730)	0.213
CAZ	2.36 (0.649–8.608)	0.153	8.32 (0.990–69.912)	**0.023**	2.44 (0.760–7.885)	0.110	4.66 (1.151–18.919)	**0.022**	2.72 (0.664–11.155)	0.133

*Note:* The *p*-value is given at 95% CI and significant at ≤ 0.05. In bold: positive association between antibiotic and the resistance gene.

Abbreviations: AMC, amoxicillin/clavuranic acid; AMX, amoxicillin; CAZ, ceftazidime; CTR, ceftriaxone; CTX, cefotaxime; CXM, cefuroxime; FOX, cefoxitin; LI, lower interval; NA, not applicable; UI, upper interval.

## Data Availability

The data used to support the findings of this study are included within the article.

## Supporting Information

Supporting material (.pdf): All raw data generated to support the results of this study (Supporting Information). S1: General information on the study population. S2: Detection of *Escherichia coli* and antibiogram. S3: Detection of virulent factors and resistance genes; S4: Image of the gel showing amplification.
