# Supplementary material for: Antibiotic Resistance Profile of Enterovirulent E. coli Isolates Harboring Broad-Spectrum Beta-Lactamase Genes in Cancer Patients at the Laquintinie Hospital in Douala, Littoral Region, Cameroon
Source: Int J Microbiol. 2025 Jan 9;2025:4224807. doi: 10.1155/ijm/4224807 (PMC11737900; doi:10.1155/ijm/4224807)
Supplement: Supporting Information — Additional supporting information can be found online in the Supporting Information section. [file 4224807.f1.pdf]

Antibiotic resistance profile of enterovirulent *E. coli* isolates harboring broad-spectrum Beta-lactamase genes in cancer patients at the Laquintine hospital in Douala, Littoral Region, and Cameroon

Michael F. Kengne[a], Armelle T. Mbaveng[a], Wiliane J. T. Marbou[a], Ousenu Karimo[a], Ballue S. T. Dadjo[a], Delano G. T. Fonjou[a], Ornella D. Tsobeng[a], and Victor Kuete[a]***

[a]Department of Biochemistry, Faculty of Science, Université de Dschang, Dschang, Cameroon

Corresponding author:
**Email: armbatsa@yahoo.fr; ORCID: https://orcid.org/0000-0003-4178-4967 (Armelle T. Mbaveng)*
***Email: kuetevictor@yahoo.fr; ORCID: http://orcid.org/0000-0002-1070-1236 (Victor Kuete)*

Other authors emails:
fmkengne@yahoo.com : *Michael F. Kengne*
ousenukarimo90@gmail.com : *Ousenu Karimo*
ballueserges@gmail.com : *Ballue S. T. Dadjo*
tsobengornella98@gmail.com : *Ornella D. Tsobeng*
fonjougiresse@gmail.com : *Delano G. T. Fonjou*
marboutakougoum@yahoo.fr : *Wiliane J. T. Marbou*

S1. General information on the study population

Code	Cancer	Sex	Age	Range age	Diarrhea	Abdominal pain	vomiting	nausea	fever	previous exposure to antibiotics	Chemotherapy
K001	yes	M	30	[30-60[	no	yes	no	yes	yes	no	no
K002	yes	M	54	[30-60[	no	yes	yes	yes	no	yes	no
K003	yes	F	50	[30-60[	no	yes	no	no	yes	no	yes
K004	yes	F	49	[30-60[	yes	yes	no	yes	yes	no	yes
K005	yes	M	31	[30-60[	yes	yes	no	no	yes	yes	yes
K006	yes	M	59	[30-60[	no	yes	no	yes	yes	no	no
K007	yes	F	67	≥60	no	yes	yes	yes	no	yes	no
K008	yes	M	38	[30-60[	no	yes	no	no	no	no	no
K009	yes	F	42	[30-60[	no	yes	no	no	no	yes	yes
K010	yes	F	43	[30-60[	no	yes	no	yes	no	no	no
K011	yes	F	41	[30-60[	yes	yes	no	no	no	yes	no
K012	yes	M	82	≥60	no	yes	no	yes	yes	yes	yes
K013	yes	F	54	[30-60[	yes	yes	no	no	yes	no	yes
K014	yes	M	18	<30	no	yes	no	yes	yes	yes	no
K015	yes	F	33	[30-60[	no	no	no	no	no	no	yes
K016	yes	M	42	[30-60[	no	yes	no	yes	no	yes	no
K017	yes	M	68	≥60	no	yes	no	no	no	no	yes
K018	yes	M	66	≥60	no	no	no	yes	no	yes	yes
K019	yes	M	70	≥60	no	yes	no	yes	yes	yes	no
K020	yes	F	43	[30-60[	no	no	no	no	no	no	yes
K021	yes	M	61	≥60	no	yes	no	no	yes	yes	no
K022	yes	M	64	≥60	no	yes	no	no	yes	yes	yes
K023	yes	M	57	[30-60[	yes	yes	yes	yes	yes	yes	yes
K024	yes	M	53	[30-60[	yes	yes	no	yes	yes	yes	no

K025	yes	F	64	≥60	no	no	no	no	no	yes	no
K026	yes	M	29	<30	no	yes	no	yes	yes	no	yes
K027	yes	M	75	≥60	no	yes	no	yes	yes	yes	no
K028	yes	F	62	≥60	no	yes	no	yes	no	yes	no
K029	yes	F	58	[30-60[	yes	yes	yes	yes	yes	yes	no
K030	yes	F	40	[30-60[	no	no	no	no	yes	no	no
K031	yes	M	65	≥60	no	yes	no	yes	yes	yes	no
K032	yes	F	50	[30-60[	no	yes	no	yes	yes	yes	no
K033	yes	F	27	<30	no	yes	no	yes	no	yes	no
K034	yes	F	38	[30-60[	no	yes	no	no	yes	no	no
K035	yes	F	43	[30-60[	no	no	no	no	no	no	yes
K036	yes	F	52	[30-60[	no	yes	no	yes	yes	yes	yes
K037	yes	F	40	[30-60[	yes	yes	yes	yes	no	no	yes
K038	yes	M	43	[30-60[	no	no	yes	yes	yes	no	yes
K039	yes	M	74	≥60	yes	yes	no	no	yes	yes	no
K040	yes	M	75	≥60	yes	yes	no	no	no	no	no
K041	yes	M	51	[30-60[	no	yes	no	no	no	yes	no
K042	yes	F	74	≥60	no	yes	no	no	yes	no	no
K043	yes	F	72	≥60	no	yes	no	no	no	yes	no
K044	yes	F	43	[30-60[	no	yes	no	no	yes	yes	no
K045	yes	M	56	[30-60[	no	yes	no	no	yes	yes	no
K046	yes	F	67	≥60	no	yes	no	no	no	no	yes
K047	yes	M	43	[30-60[	no	yes	no	no	no	yes	yes
K048	yes	F	55	[30-60[	no	no	no	yes	no	yes	yes
K049	yes	F	50	[30-60[	no	yes	no	yes	yes	no	no
K050	yes	M	50	[30-60[	no	yes	no	no	no	yes	no
K051	yes	F	42	[30-60[	no	yes	no	no	no	no	no

K052	yes	F	45	[30-60[	no	no	no	no	no	yes	no
K053	yes	F	47	[30-60[	no	yes	no	yes	yes	yes	no
K054	yes	F	63	≥60	no	yes	no	no	yes	yes	no
K055	yes	F	10	<30	no	yes	no	yes	yes	yes	yes
K056	yes	F	69	≥60	no	no	no	yes	yes	yes	yes
K057	yes	M	66	≥60	no	no	yes	yes	yes	no	yes
K058	yes	F	59	[30-60[	no	yes	no	no	yes	yes	yes
K059	yes	M	60	≥60	no	yes	no	yes	yes	no	no
K060	yes	F	56	[30-60[	yes	yes	no	yes	yes	yes	yes
K061	yes	F	40	[30-60[	no	yes	no	yes	yes	no	no
K062	yes	F	38	[30-60[	no	yes	yes	yes	yes	yes	no
K063	yes	M	78	≥60	no	no	no	no	no	no	yes
K064	yes	F	60	≥60	no	yes	no	yes	yes	yes	yes
K065	yes	M	68	≥60	no	yes	yes	yes	yes	yes	no
K066	yes	M	77	≥60	no	no	no	no	no	yes	yes
K067	yes	M	39	[30-60[	no	yes	yes	yes	yes	yes	no
K068	yes	F	36	[30-60[	no	yes	yes	yes	no	yes	yes
K069	yes	M	34	[30-60[	no	yes	no	no	no	no	no
K070	yes	M	29	<30	yes	no	yes	no	yes	yes	no
K071	yes	M	55	[30-60[	no	yes	no	no	no	no	no
K072	yes	F	57	[30-60[	no	yes	no	no	yes	yes	no
K073	yes	F	41	[30-60[	no	yes	no	no	no	no	no
K074	yes	F	23	<30	no	yes	no	no	yes	yes	no
K075	yes	F	65	≥60	no	yes	no	yes	no	yes	yes
K076	yes	F	75	≥60	no	yes	no	yes	yes	no	no
K077	yes	M	47	[30-60[	yes	yes	no	yes	yes	yes	yes
K078	yes	M	57	[30-60[	no	yes	yes	yes	yes	yes	no

K079	yes	F	50	[30-60[	no	yes	no	no	no	no	yes
K080	yes	F	41	[30-60[	no	no	no	no	no	yes	yes
K081	yes	F	60	≥60	yes	no	no	no	no	yes	no
K082	yes	F	51	[30-60[	no	no	no	no	no	yes	no
K083	yes	F	49	[30-60[	yes	yes	no	no	yes	yes	yes
K084	yes	M	70	≥60	no	yes	no	no	no	no	yes
K085	yes	F	44	[30-60[	no	yes	no	yes	no	yes	no
K086	yes	M	70	≥60	no	yes	no	no	yes	yes	no
K087	yes	F	67	≥60	yes	no	no	no	no	no	yes
K088	yes	F	44	[30-60[	no	no	no	no	no	no	yes
K089	yes	M	75	≥60	no	yes	no	no	no	yes	no
K090	yes	M	50	[30-60[	no	yes	no	yes	yes	yes	no
K091	yes	M	58	[30-60[	no	yes	no	yes	yes	yes	no
K092	yes	F	37	[30-60[	no	yes	no	no	no	no	no
K093	yes	F	48	[30-60[	no	yes	no	no	no	no	yes
K094	yes	M	33	[30-60[	no	no	no	no	no	no	no
K095	yes	F	50	[30-60[	no	yes	no	no	yes	yes	yes
K096	yes	M	69	≥60	yes	yes	no	no	no	yes	yes
K097	yes	F	48	[30-60[	no	no	no	no	no	no	yes
K098	yes	F	15	<30	yes	yes	no	yes	yes	no	yes
K099	yes	M	58	[30-60[	yes	yes	no	yes	yes	no	yes
K100	yes	F	52	[30-60[	no	no	no	no	yes	yes	yes
K101	yes	F	29	<30	no	yes	no	no	no	no	yes
K102	yes	M	13	<30	yes	no	no	no	no	no	yes
K103	yes	M	32	[30-60[	no	no	no	no	no	yes	yes
K104	yes	F	54	[30-60[	yes	yes	no	no	no	yes	no
K105	yes	F	56	[30-60[	no	no	no	no	no	yes	yes

K106	yes	F	41	[30-60[	no	yes	no	no	no	yes	yes
K107	yes	M	11	<30	no	yes	no	no	no	yes	yes
K108	yes	F	44	[30-60[	no	yes	no	no	no	yes	yes
K109	yes	F	43	[30-60[	no	no	yes	yes	yes	no	yes
K110	yes	F	38	[30-60[	no	yes	no	no	no	yes	yes
K111	yes	F	12	<30	yes	no	no	no	no	no	yes
K112	yes	F	41	[30-60[	yes	yes	no	yes	yes	no	yes
K113	yes	M	57	[30-60[	no	yes	no	no	no	no	yes
K114	yes	F	38	[30-60[	no	yes	no	no	no	no	yes
K115	yes	F	54	[30-60[	no	yes	no	no	no	no	yes
K116	yes	F	44	[30-60[	no	no	yes	yes	yes	yes	yes
K117	yes	F	50	[30-60[	yes	no	no	no	yes	yes	yes
K118	yes	M	71	≥60	no	yes	no	no	no	no	yes
K119	yes	F	68	≥60	yes	yes	yes	no	yes	no	yes
K120	yes	F	61	≥60	yes	yes	yes	no	yes	yes	yes
K121	yes	F	60	≥60	yes	yes	yes	yes	yes	no	yes
K122	yes	F	49	[30-60[	no	no	no	no	no	no	yes
K123	yes	F	39	[30-60[	no	yes	no	no	no	no	yes
K124	yes	M	37	[30-60[	no	no	no	no	no	no	yes
K125	yes	F	64	≥60	no	no	no	no	no	no	yes
K126	yes	F	36	[30-60[	yes	yes	no	no	no	yes	yes
K127	yes	F	45	[30-60[	no	yes	no	no	yes	no	yes
K128	yes	F	50	[30-60[	no	yes	no	no	no	yes	yes
K129	yes	F	30	[30-60[	yes	yes	no	no	yes	no	yes
K130	yes	F	45	[30-60[	no	yes	no	yes	yes	yes	no
K131	yes	F	47	[30-60[	no	no	no	no	no	yes	no
K132	yes	F	76	≥60	no	yes	yes	yes	yes	yes	no

K133	yes	F	40	[30-60[	yes	yes	yes	yes	yes	no	no
K134	yes	F	27	<30	no	no	no	yes	no	yes	no
K135	yes	M	80	≥60	yes	no	no	no	yes	yes	no
K136	yes	F	55	[30-60[	no	yes	no	yes	yes	yes	no
K137	yes	M	11	<30	yes	yes	no	no	no	no	yes
K138	yes	F	50	[30-60[	no	yes	no	no	no	yes	yes
K139	yes	M	10	<30	yes	yes	yes	yes	yes	yes	yes
K140	yes	M	63	≥60	no	yes	no	no	no	no	yes
K141	yes	F	19	<30	no	no	no	nn	no	no	yes
K142	yes	M	42	[30-60[	yes	no	yes	yes	yes	no	yes
K143	yes	M	32	[30-60[	no	no	no	no	no	no	yes
K144	yes	M	46	[30-60[	yes	yes	no	no	yes	yes	yes
K145	yes	F	54	[30-60[	no	yes	no	yes	yes	yes	yes
K146	yes	F	43	[30-60[	no	yes	no	no	yes	yes	no
K147	yes	M	58	[30-60[	yes	yes	yes	yes	yes	yes	yes
K148	yes	F	72	≥60	no	yes	no	no	no	no	yes
K149	yes	F	49	[30-60[	no	yes	yes	yes	yes	yes	yes
K150	yes	F	79	≥60	yes	yes	yes	yes	yes	yes	yes
K151	yes	F	62	≥60	no	yes	yes	yes	yes	yes	no
K152	yes	F	66	≥60	yes	no	yes	yes	yes	yes	yes
K153	yes	F	66	≥60	no	no	no	no	no	yes	no
K154	yes	F	51	[30-60[	no	yes	no	no	no	yes	yes
K155	yes	F	46	[30-60[	no	yes	no	no	yes	no	yes
K156	yes	F	60	≥60	no	yes	no	no	no	yes	yes
K157	yes	F	68	≥60	yes	yes	no	no	yes	yes	yes
K158	yes	M	61	≥60	no	yes	no	no	no	yes	yes
K159	yes	M	45	[30-60[	yes	yes	no	no	no	yes	yes

K160	yes	F	47	[30-60[	no	yes	no	no	no	yes	yes
K161	yes	F	32	[30-60[	no	no	no	no	no	yes	yes
K162	yes	F	54	[30-60[	no	yes	yes	yes	no	yes	yes
K163	yes	F	64	≥60	no	yes	no	no	no	no	yes
K164	yes	F	48	[30-60[	no	yes	yes	yes	yes	yes	yes
K165	yes	M	43	[30-60[	yes	yes	no	yes	yes	no	yes
K166	yes	M	64	≥60	no	yes	no	yes	yes	yes	yes
K167	yes	F	60	≥60	no	yes	yes	yes	yes	yes	no
K168	yes	M	56	[30-60[	yes	no	yes	yes	no	yes	yes
K169	yes	M	47	[30-60[	no	yes	yes	yes	yes	no	yes
K170	yes	F	56	[30-60[	no	yes	no	no	yes	no	yes
K171	yes	F	39	[30-60[	no	yes	no	no	no	no	no
K172	yes	F	31	[30-60[	yes	yes	no	yes	yes	yes	no
K173	yes	M	57	[30-60[	yes	yes	no	no	no	yes	yes
K174	yes	M	54	[30-60[	no	yes	yes	yes	yes	yes	yes
K175	yes	M	28	<30	yes	yes	no	no	yes	yes	no
K176	yes	M	45	[30-60[	no	yes	no	no	no	yes	no
K177	yes	M	62	≥60	yes	yes	no	yes	yes	yes	no
K178	yes	M	62	≥60	yes	yes	no	yes	no	no	yes
K179	yes	M	55	[30-60[	no	no	no	no	yes	no	yes
K180	yes	F	46	[30-60[	no	yes	no	no	no	no	yes
K181	yes	M	42	[30-60[	no	no	no	no	yes	no	yes
K182	yes	M	72	≥60	no	yes	no	no	yes	no	yes
K183	yes	F	45	[30-60[	yes	yes	no	yes	yes	no	no
K184	yes	M	69	≥60	no	yes	no	no	no	yes	yes
K185	yes	F	36	[30-60[	no	no	no	no	no	no	yes
K186	yes	M	41	[30-60[	no	yes	yes	yes	yes	yes	yes

K187	yes	F	47	[30-60[	yes	yes	yes	yes	yes	yes	yes
K188	yes	M	67	≥60	no	yes	yes	yes	yes	yes	no
K189	yes	F	63	≥60	yes	yes	no	yes	no	yes	no
K190	yes	F	66	≥60	no	yes	no	no	yes	yes	no
K191	yes	M	28	<30	no	no	no	no	no	yes	yes
K192	yes	M	54	[30-60[	no	yes	no	yes	yes	yes	yes
K193	yes	M	41	[30-60[	no	yes	no	no	no	no	no
K194	yes	F	36	[30-60[	no	yes	no	no	no	no	yes
K195	yes	M	48	[30-60[	yes	no	no	no	no	yes	yes
K196	yes	F	64	≥60	yes	yes	no	yes	yes	yes	yes
K197	yes	M	60	≥60	no	yes	no	no	no	no	yes
K198	yes	M	45	[30-60[	no	yes	no	no	no	no	yes
K199	yes	M	41	[30-60[	no	yes	no	yes	yes	yes	no
K200	yes	F	54	[30-60[	no	no	no	no	no	yes	yes
K201	yes	M	37	[30-60[	yes	no	no	no	no	yes	yes
K202	yes	M	39	[30-60[	no	yes	no	yes	yes	yes	no
K203	yes	M	43	[30-60[	yes	yes	no	yes	yes	yes	no
K204	yes	F	60	≥60	no	no	no	no	no	no	yes
K205	yes	F	43	[30-60[	yes	yes	no	yes	no	no	no
K206	yes	F	40	[30-60[	yes	no	no	no	no	no	yes
K207	yes	M	44	[30-60[	no	no	no	no	yes	no	yes
K208	yes	F	61	≥60	no	yes	yes	no	no	yes	yes
K209	yes	F	56	[30-60[	no	yes	no	no	no	yes	yes
K210	yes	F	31	[30-60[	no	yes	yes	yes	yes	yes	no
K211	yes	F	62	≥60	no	no	yes	no	yes	no	yes
K212	yes	M	52	[30-60[	yes	no	yes	no	yes	no	yes
K213	yes	F	63	≥60	no	yes	no	no	no	no	yes

K214	yes	F	39	[30-60[	no	yes	no	no	yes	yes	yes
K215	yes	F	45	[30-60[	no	yes	no	yes	yes	no	yes
K216	yes	M	44	[30-60[	no	yes	no	yes	yes	yes	yes
K217	yes	F	31	[30-60[	yes	yes	no	no	no	yes	yes
K218	yes	F	47	[30-60[	no	no	no	no	no	yes	yes
K219	yes	F	36	[30-60[	no	yes	no	yes	yes	no	yes
K220	yes	F	28	<30	no	yes	yes	yes	yes	yes	no
K221	yes	F	47	[30-60[	no	yes	no	yes	no	yes	no
K222	yes	F	42	[30-60[	yes	yes	yes	no	no	yes	yes
K223	yes	F	72	≥60	no	yes	yes	yes	no	yes	no
K224	yes	F	49	[30-60[	no	yes	yes	yes	yes	yes	no
K225	yes	F	36	[30-60[	no	yes	no	no	no	yes	yes
K226	yes	F	45	[30-60[	no	yes	yes	yes	yes	no	yes
K227	yes	F	75	≥60	yes	no	no	no	no	yes	no
K228	yes	F	29	<30	yes	no	no	no	no	no	no
K229	yes	M	46	[30-60[	no	no	yes	no	no	yes	yes
K230	yes	F	45	[30-60[	no	yes	yes	no	yes	yes	yes
K231	yes	F	54	[30-60[	no	yes	no	yes	no	yes	yes
K232	yes	F	47	[30-60[	no	no	no	yes	no	yes	yes
K233	yes	F	49	[30-60[	no	yes	yes	yes	yes	yes	no
K234	yes	F	36	[30-60[	no	yes	yes	no	yes	yes	no
K235	yes	F	40	[30-60[	no	yes	no	yes	yes	yes	no
K236	yes	M	81	≥60	no	yes	yes	yes	yes	yes	no
K237	yes	F	61	≥60	no	yes	yes	no	no	no	no
K238	yes	F	68	≥60	no	yes	no	yes	yes	yes	no
K239	yes	F	20	<30	no	yes	no	yes	yes	yes	no
K240	yes	M	80	≥60	no	yes	yes	yes	yes	no	no

K241	yes	F	56	[30-60[	yes	no	no	yes	yes	yes	yes
K242	yes	F	39	[30-60[	no	yes	no	no	yes	yes	yes
K243	yes	F	45	[30-60[	no	yes	yes	yes	yes	yes	yes
K244	yes	F	38	[30-60[	no	yes	no	no	no	yes	yes
K245	yes	F	33	[30-60[	no	yes	yes	yes	yes	no	yes
K246	yes	F	76	≥60	no	yes	no	no	yes	yes	yes
K247	yes	F	62	≥60	yes	yes	no	no	no	yes	no
K248	yes	F	44	[30-60[	no	yes	no	yes	yes	yes	no
K249	yes	F	43	[30-60[	no	no	no	no	no	yes	yes
K250	yes	F	50	[30-60[	yes	yes	yes	yes	yes	yes	no
K251	yes	F	45	[30-60[	no	yes	no	no	no	yes	yes
K252	yes	M	14	<30	yes	yes	no	no	no	no	yes
K253	yes	F	43	[30-60[	no	yes	no	no	no	yes	no
K254	yes	F	61	≥60	yes	no	no	no	yes	yes	yes
K255	yes	M	71	≥60	no	yes	no	yes	yes	yes	yes
K256	yes	M	30	[30-60[	no	yes	yes	yes	yes	no	no
K257	yes	M	61	≥60	no	yes	yes	yes	yes	yes	no
K258	yes	F	44	[30-60[	no	yes	yes	yes	yes	yes	no
K259	yes	F	62	≥60	no	yes	yes	yes	yes	yes	yes
K260	yes	F	46	[30-60[	no	yes	no	yes	yes	no	yes
K261	yes	F	47	[30-60[	no	yes	no	no	no	yes	yes
K262	yes	M	47	[30-60[	no	yes	yes	yes	yes	yes	yes
K263	yes	F	35	[30-60[	yes	yes	no	no	yes	yes	yes
K264	yes	M	45	[30-60[	no	yes	no	no	no	no	no
K265	yes	F	43	[30-60[	no	yes	yes	yes	yes	yes	yes
K266	yes	F	46	[30-60[	no	yes	yes	no	yes	yes	yes
K267	yes	F	54	[30-60[	yes	yes	yes	yes	yes	no	yes

K268	yes	F	69	≥60	no	yes	yes	yes	no	yes	yes
K269	yes	F	71	≥60	no	yes	no	yes	yes	yes	yes
K270	yes	F	69	≥60	yes	yes	yes	yes	yes	yes	yes
K271	yes	F	58	[30-60[	no	yes	yes	no	yes	yes	yes
K272	yes	F	46	[30-60[	no	yes	no	yes	yes	yes	no
K273	yes	F	52	[30-60[	no	yes	no	yes	yes	no	no
K274	yes	F	39	[30-60[	no	yes	yes	yes	yes	no	yes
K275	yes	F	52	[30-60[	yes	yes	no	yes	yes	yes	yes
K276	yes	F	48	[30-60[	no	no	no	no	no	yes	yes
K277	yes	F	53	[30-60[	no	yes	no	yes	yes	yes	yes
K278	yes	F	58	[30-60[	no	yes	yes	yes	yes	yes	yes
K279	yes	F	45	[30-60[	no	yes	yes	yes	yes	yes	yes
K280	yes	M	41	[30-60[	yes	yes	yes	yes	yes	yes	no
K281	yes	F	49	[30-60[	no	yes	no	no	yes	yes	yes
K282	yes	F	53	[30-60[	yes	yes	no	yes	no	yes	no
K283	yes	F	60	≥60	no	yes	no	no	no	yes	no
K284	yes	F	46	[30-60[	yes	yes	no	no	yes	yes	no
K285	yes	F	57	[30-60[	no	yes	no	no	no	yes	no
K286	yes	M	47	[30-60[	yes	yes	no	yes	yes	yes	no
K287	yes	M	56	[30-60[	yes	yes	yes	yes	yes	yes	yes
K288	yes	F	26	<30	no	yes	no	no	no	yes	yes
K289	yes	M	28	<30	no	yes	no	no	no	no	yes
K290	yes	F	60	≥60	yes	no	no	no	no	yes	yes
K291	yes	F	32	[30-60[	no	yes	yes	yes	yes	yes	yes
K292	yes	M	27	<30	yes	yes	no	no	yes	yes	yes
K293	yes	M	42	[30-60[	yes	yes	no	yes	yes	yes	no
K294	yes	F	41	[30-60[	no	yes	yes	no	no	yes	yes

K295	yes	M	28	<30	yes	yes	no	no	no	yes	yes
K296	yes	F	48	[30-60[	yes	no	no	yes	yes	yes	yes
K297	yes	F	68	≥60	no	yes	no	no	no	yes	yes
K298	yes	F	64	≥60	no	yes	no	no	no	yes	yes
K299	yes	M	59	[30-60[	no	yes	yes	yes	yes	yes	no
K300	yes	M	52	[30-60[	no	yes	no	yes	yes	yes	no
K301	yes	M	25	<30	yes	yes	no	no	yes	no	no
K302	yes	F	75	≥60	no	yes	no	no	no	yes	no
K303	yes	F	49	[30-60[	no	yes	no	no	no	yes	no
K304	yes	F	41	[30-60[	no	yes	no	no	no	yes	yes
K305	yes	F	63	≥60	yes	yes	yes	yes	yes	no	yes
K306	yes	M	16	<30	no	no	yes	yes	yes	yes	yes
K307	yes	F	50	[30-60[	yes	yes	no	yes	no	yes	yes
D001	no	M	45	[30-60[	yes	no	no	no	no	no	
D002	no	F	64	≥60	yes	no	no	no	no	no	
D003	no	F	52	[30-60[	yes	yes	no	no	no	yes	
D004	no	M	42	[30-60[	yes	yes	no	no	no	no	
D005	no	M	69	≥60	no	no	no	no	no	yes	
D006	no	F	34	[30-60[	yes	yes	yes	yes	yes	no	
D007	no	M	49	[30-60[	no	no	no	no	no	no	
D008	no	F	44	[30-60[	no	yes	yes	no	no	yes	
D009	no	M	77	≥60	no	no	no	yes	yes	yes	
D010	no	M	10	<30	yes	yes	no	no	no	yes	
D011	no	M	40	[30-60[	no	no	yes	no	no	yes	
D012	no	M	31	[30-60[	no	no	no	no	no	no	
D013	no	F	38	[30-60[	yes	no	no	yes	no	no	
D014	no	F	55	[30-60[	no	yes	yes	no	yes	no	

D015	no	F	41	[30-60[	no	yes	no	yes	no	yes	
D016	no	F	20	<30	no	no	no	no	no	yes	
D017	no	F	61	≥60	no	no	no	no	no	no	
D018	no	F	23	<30	yes	yes	yes	yes	yes	no	
D019	no	F	53	[30-60[	no	yes	no	no	no	no	
D020	no	F	34	[30-60[	no	yes	no	no	no	no	
D021	no	F	36	[30-60[	no	yes	no	no	no	no	
D022	no	M	10	<30	yes	no	no	yes	no	no	
D023	no	M	28	<30	yes	no	yes	yes	yes	no	
D024	no	F	22	<30	no	yes	no	no	no	no	
D025	no	F	38	[30-60[	yes	yes	no	no	no	no	
D026	no	F	20	<30	yes	no	no	yes	yes	no	
D027	no	M	35	[30-60[	yes	no	no	no	yes	no	
D028	no	F	40	[30-60[	no	yes	no	no	no	yes	
D029	no	F	53	[30-60[	no	yes	no	no	no	no	
D030	no	F	45	[30-60[	yes	no	yes	yes	yes	no	
D031	no	F	36	[30-60[	yes	no	yes	yes	no	yes	
D032	no	M	63	≥60	no	no	no	no	no	no	
D033	no	F	22	<30	no	yes	no	yes	yes	yes	
D034	no	F	38	[30-60[	yes	yes	yes	yes	yes	yes	
D035	no	M	57	[30-60[	yes	no	no	no	no	no	
D036	no	F	20	<30	no	no	yes	yes	no	no	
D037	no	F	54	[30-60[	no	yes	no	no	no	no	
D038	no	F	27	<30	no	yes	yes	no	no	yes	
D039	no	F	25	<30	no	yes	no	yes	yes	no	
D040	no	F	58	[30-60[	yes	yes	yes	no	yes	no	
D041	no	M	27	<30	yes	no	no	no	no	yes	

D042	no	F	66	≥60	no	no	no	no	yes	no	
D043	no	M	10	<30	no	yes	yes	no	yes	no	
D044	no	F	37	[30-60[	no	yes	no	no	no	no	
D045	no	F	78	≥60	no	no	yes	no	yes	no	
D046	no	M	63	≥60	no	yes	no	no	no	yes	
D047	no	M	43	[30-60[	yes	no	yes	yes	yes	no	
D048	no	F	63	≥60	no	yes	no	yes	no	yes	
D049	no	M	52	[30-60[	no	no	no	no	yes	no	
D050	no	F	23	<30	no	yes	yes	yes	no	no	
D051	no	M	42	[30-60[	no	no	no	no	no	no	
D052	no	F	23	<30	yes	yes	yes	no	yes	no	
D053	no	F	54	[30-60[	no	yes	no	yes	no	no	
D054	no	F	31	[30-60[	yes	no	no	yes	no	yes	
D055	no	M	44	[30-60[	yes	no	no	no	yes	yes	
D056	no	M	43	[30-60[	no	no	no	yes	no	no	
D057	no	M	40	[30-60[	yes	no	no	no	yes	no	
D058	no	M	47	[30-60[	yes	no	no	no	yes	no	
D059	no	M	10	<30	no	no	no	yes	yes	yes	
D060	no	M	41	[30-60[	yes	yes	yes	no	no	no	
D061	no	M	37	[30-60[	no	no	no	yes	yes	no	
D062	no	F	17	<30	no	yes	no	no	no	no	
D063	no	F	30	[30-60[	no	yes	no	yes	yes	no	
D064	no	F	38	[30-60[	yes	no	no	yes	yes	no	
D065	no	M	30	[30-60[	no	yes	yes	yes	no	yes	
D066	no	F	33	[30-60[	no	yes	yes	no	yes	no	
D067	no	F	84	≥60	no	yes	yes	yes	no	yes	
D068	no	F	38	[30-60[	no	yes	no	yes	yes	no	

D069	no	M	48	[30-60[	yes	yes	no	no	yes	yes	
D070	no	F	39	[30-60[	no	no	no	yes	no	no	
D071	no	F	44	[30-60[	no	yes	no	no	yes	no	
D072	no	F	34	[30-60[	yes	no	no	yes	no	no	
D073	no	F	10	<30	no	yes	yes	yes	no	yes	
D074	no	F	42	[30-60[	no	yes	no	yes	no	no	
D075	no	F	36	[30-60[	no	yes	no	yes	no	yes	
D076	no	M	29	<30	no	yes	yes	no	no	no	
D077	no	F	20	<30	yes	no	yes	no	yes	no	
D078	no	M	29	<30	yes	no	yes	no	yes	yes	
D079	no	F	10	<30	no	yes	no	no	yes	no	
D080	no	M	81	≥60	yes	no	yes	no	no	no	
D081	no	F	29	<30	yes	no	yes	yes	yes	yes	
D082	no	F	51	[30-60[	no	yes	yes	no	no	no	
D083	no	M	35	[30-60[	no	yes	no	yes	yes	no	
D084	no	F	51	[30-60[	no	yes	no	no	no	no	
D085	no	F	42	[30-60[	no	no	yes	no	no	no	
D086	no	M	64	≥60	no	yes	no	no	no	no	
D087	no	M	56	[30-60[	no	yes	no	no	no	yes	
D088	no	F	33	[30-60[	no	no	no	no	yes	no	
D089	no	M	34	[30-60[	yes	yes	no	yes	no	no	
D090	no	M	58	[30-60[	yes	no	yes	no	no	yes	
D091	no	M	54	[30-60[	no	yes	no	no	no	no	
D092	no	M	49	[30-60[	no	yes	no	yes	no	no	
D093	no	M	48	[30-60[	yes	yes	no	yes	no	no	
D094	no	M	52	[30-60[	yes	no	no	yes	yes	no	
D095	no	F	34	[30-60[	no	no	yes	yes	no	no	

D096	no	F	29	<30	yes	no	no	no	yes	no	
D097	no	M	25	<30	no	no	yes	yes	no	no	
D098	no	F	55	[30-60[	yes	yes	no	yes	yes	no	
D099	no	M	41	[30-60[	yes	no	no	yes	yes	yes	
D100	no	F	48	[30-60[	yes	no	yes	no	no	yes	
D101	no	F	42	[30-60[	yes	yes	yes	no	yes	yes	
D102	no	F	45	[30-60[	yes	yes	no	yes	yes	no	
D103	no	F	23	<30	no	no	yes	no	no	no	
D104	no	M	47	[30-60[	yes	no	no	yes	yes	no	
D105	no	M	15	<30	no	yes	no	no	no	no	
D106	no	F	68	≥60	yes	no	yes	yes	no	no	
D107	no	M	39	[30-60[	no	yes	yes	no	yes	no	
D108	no	M	47	[30-60[	no	yes	no	yes	yes	no	
D109	no	F	36	[30-60[	yes	yes	no	no	yes	no	
D110	no	M	45	[30-60[	no	yes	yes	yes	no	no	
D111	no	F	42	[30-60[	no	yes	yes	no	no	yes	
D112	no	F	20	<30	no	yes	no	no	yes	yes	
D113	no	F	23	<30	yes	no	no	no	no	no	
D114	no	M	47	[30-60[	yes	no	no	no	no	no	
D115	no	M	20	<30	no	yes	no	no	yes	yes	
D116	no	F	68	≥60	no	yes	yes	no	no	no	
D117	no	M	39	[30-60[	no	yes	yes	no	no	no	
D118	no	M	47	[30-60[	yes	no	no	no	no	no	
D119	no	F	36	[30-60[	no	yes	yes	no	no	no	
D120	no	M	32	[30-60[	yes	no	yes	no	no	yes	
D121	no	F	80	≥60	no	yes	no	yes	no	yes	
D122	no	F	53	[30-60[	no	no	yes	no	yes	no	

D123	no	F	29	<30	no	no	no	no	yes	no	
D124	no	M	66	≥60	no	yes	no	no	yes	no	
D125	no	M	71	≥60	yes	yes	yes	yes	no	no	
D126	no	M	42	[30-60[	no	yes	yes	yes	yes	yes	
D127	no	F	28	<30	yes	yes	no	no	yes	yes	
D128	no	M	33	[30-60[	no	no	yes	no	yes	no	
D129	no	F	72	≥60	yes	no	no	no	no	yes	
D130	no	F	60	≥60	no	yes	yes	no	no	no	
D131	no	F	70	≥60	no	yes	no	no	no	no	
D132	no	M	29	<30	no	yes	yes	yes	yes	no	
D133	no	F	35	[30-60[	no	yes	no	yes	yes	yes	
D134	no	M	46	[30-60[	no	no	no	yes	yes	no	
D135	no	M	39	[30-60[	yes	yes	yes	no	no	no	
D136	no	F	65	≥60	no	yes	no	yes	yes	no	
D137	no	M	44	[30-60[	no	no	no	yes	no	no	
D138	no	F	65	≥60	no	no	yes	yes	no	no	
D139	no	F	44	[30-60[	no	no	yes	no	no	no	
D140	no	F	64	≥60	no	yes	yes	yes	yes	no	
D141	no	F	56	[30-60[	yes	no	yes	yes	yes	no	
D142	no	F	66	≥60	no	yes	no	no	no	no	
D143	no	M	34	[30-60[	yes	no	yes	no	no	no	
D144	no	F	58	[30-60[	no	no	yes	no	yes	no	
D145	no	F	54	[30-60[	no	yes	yes	yes	yes	yes	
D146	no	F	48	[30-60[	no	yes	no	yes	no	yes	
D147	no	M	52	[30-60[	no	no	yes	no	no	no	
D148	no	M	34	[30-60[	yes	no	no	no	yes	yes	
D149	no	F	29	<30	no	yes	no	no	no	no	

D150	no	F	38	[30-60[	no	yes	no	yes	yes	yes	
D151	no	F	36	[30-60[	no	yes	yes	no	yes	yes	
D152	no	M	10	<30	no	yes	no	yes	no	yes	
D153	no	M	28	<30	no	no	no	no	no	no	
D154	no	F	22	<30	no	no	no	no	no	no	
D155	no	F	38	[30-60[	yes	yes	yes	yes	yes	no	
D156	no	F	20	<30	no	yes	no	no	no	no	
D157	no	M	35	[30-60[	no	yes	no	no	no	no	
D158	no	F	40	[30-60[	no	yes	no	no	no	yes	
D159	no	F	53	[30-60[	yes	no	no	yes	no	yes	
D160	no	F	45	[30-60[	no	no	yes	yes	yes	yes	
D161	no	F	36	[30-60[	no	yes	no	no	no	no	
D162	no	M	63	≥60	yes	yes	no	no	no	no	
D163	no	F	22	<30	yes	no	no	yes	yes	no	
D164	no	F	38	[30-60[	yes	no	no	no	yes	no	
D165	no	M	57	[30-60[	no	yes	no	no	no	no	
D166	no	F	20	<30	no	yes	no	no	no	no	
D167	no	F	54	[30-60[	yes	no	yes	yes	yes	no	
D168	no	F	27	<30	yes	no	yes	yes	no	no	
D169	no	F	25	<30	no	no	no	no	no	no	
D170	no	F	58	[30-60[	no	yes	no	yes	yes	no	
D171	no	M	27	<30	no	yes	yes	yes	yes	no	
D172	no	F	66	≥60	no	no	no	no	no	no	
D173	no	M	10	<30	no	no	yes	yes	no	no	
D174	no	F	37	[30-60[	no	yes	no	no	no	yes	
D175	no	F	78	≥60	no	yes	yes	no	no	no	
D176	no	M	63	≥60	no	yes	no	yes	yes	no	

D177	no	M	43	[30-60[	no	yes	yes	no	yes	no	
D178	no	F	63	≥60	yes	no	no	no	no	yes	
D179	no	M	52	[30-60[	no	no	no	no	yes	no	
D180	no	F	23	<30	no	yes	yes	no	yes	yes	
D181	no	M	42	[30-60[	no	yes	no	no	no	no	
D182	no	F	23	<30	no	no	yes	no	yes	no	
D183	no	F	54	[30-60[	no	yes	no	no	no	no	
D184	no	F	31	[30-60[	yes	no	yes	yes	yes	no	
D185	no	M	44	[30-60[	no	yes	no	yes	no	no	
D186	no	M	43	[30-60[	yes	no	no	no	yes	no	
D187	no	M	40	[30-60[	no	yes	yes	yes	no	no	
D188	no	M	47	[30-60[	no	no	no	no	no	yes	
D189	no	M	10	<30	yes	yes	yes	no	yes	no	
D190	no	M	41	[30-60[	no	yes	no	yes	no	yes	
D191	no	M	37	[30-60[	yes	no	no	yes	no	no	
D192	no	F	17	<30	yes	no	no	no	yes	no	
D193	no	F	30	[30-60[	yes	no	no	yes	no	no	
D194	no	F	38	[30-60[	yes	no	no	no	yes	no	
D195	no	M	30	[30-60[	yes	no	no	no	yes	yes	
D196	no	F	33	[30-60[	no	no	no	yes	yes	no	
D197	no	F	84	≥60	no	yes	yes	no	no	no	
D198	no	F	38	[30-60[	no	no	no	yes	yes	no	
D199	no	M	48	[30-60[	yes	no	no	yes	no	no	
D200	no	F	39	[30-60[	no	yes	yes	yes	yes	yes	

Legend: F: female; M: male

S2. Detection of Escherichia coli and antibiogram

Code	Eschericia coli infection	IMP	AMX	AMC	CAZ	FOX	CTX	CXM	COT	ERY	AMK	GEN	CIP	OFX	NAL	CTR	COL	PRL	TET	VAN	NIT	ATM	FOS	MDR
K001	YES	S	R	S	R	R	R	I	R	R	S	S	R	R	S	S	S	R	R	R	S	S	S	yes
K002	NO																							
K003	YES	S	R	S	R	S	R	R	S	S	S	S	S	S	R	R	S	R	R	R	S	S	S	yes
K004	YES	S	R	R	R	S	R	R	S	R	S	S	R	R	R	R	I	R	R	R	S	S	S	yes
K005	YES	S	R	S	R	R	R	I	S	R	S	S	R	R	S	R	R	R	R	R	R	s	s	yes
K006	NO																							
K007	YES	S	R	R	R	R	R	R	S	S	S	S	R	R	S	S	S	R	R	R	S	S	S	yes
K008	NO																							
K009	NO																							
K010	NO																							
K011	NO																							
K012	YES	S	R	R	R	S	R	R	S	I	S	S	R	R	R	R	S	R	R	R	S	R	R	yes
K013	YES	S	R	R	R	R	R	I	S	I	S	S	S	S	S	S	S	R	R	R	S	R	R	yes
K014	YES	S	R	R	R	S	R	R	S	R	S	S	S	S	S	S	R	R	S	R	S	R	R	yes
K015	NO																							
K016	YES	S	R	S	S	S	S	S	S	I	S	S	S	S	S	S	S	R	R	R	S	S	S	no
K017	NO																							
K018	NO																							
K019	NO																							
K020	YES	S	R	S	S	S	R	S	S	R	S	S	S	S	R	R	R	R	R	R	S	S	R	yes
K021	NO																							
K022	YES	S	R	S	S	S	S	S	S	S	S	S	S	S	S	S	R	R	R	R	S	S	S	no
K023	YES	S	R	S	R	R	R	R	R	R	S	S	R	R	R	R	R	R	R	R	R	R	R	yes
K024	NO																							

K025	NO																						
K026	YES	S	R	R	R	R	R	R	S	R	S	S	R	R	R	R	R	R	R	R	S	R	R	yes
K027	YES	S	R	S	S	S	S	S	S	S	S	S	R	R	R	R	R	R	R	R	S	S	R	no
K028	NO																						
K029	YES	S	R	R	R	S	R	R	S	R	S	R	R	R	R	R	R	R	R	R	S	R	S	yes
K030	NO																						
K031	NO																						
K032	NO																						
K033	YES	S	R	R	R	R	R	R	S	R	S	S	R	R	R	R	R	R	R	R	S	S	R	yes
K034	YES	S	R	R	R	R	R	R	S	R	S	S	R	R	R	R	R	R	R	R	S	R	R	yes
K035	YES	S	R	R	R	R	R	R	S	R	S	S	R	R	S	S	R	R	S	R	S	S	R	yes
K036	YES	S	R	R	S	S	S	R	R	R	S	S	R	R	R	R	R	R	R	R	S	S	S	yes
K037	NO																						
K038	NO																						
K039	YES	S	R	S	S	S	R	R	S	S	S	S	R	R	S	S	R	R	R	R	S	S	R	no
K040	YES	S	R	R	R	R	R	R	R	R	S	R	R	R	R	R	R	R	R	R	R	R	R	yes
K041	NO																						
K042	YES	S	R	R	R	R	R	R	R	R	S	S	R	R	R	R	S	R	R	R	S	R	S	yes
K043	NO																						
K044	NO																						
K045	NO																						
K046	NO																						
K047	YES	S	S	S	S	S	S	S	S	S	S	S	S	S	S	S	R	S	R	S	S	S	S	no
K048	YES	S	R	R	I	R	R	R	S	S	S	S	S	S	S	S	R	R	R	R	S	R	R	yes
K049	NO																						
K050	YES	S	R	R	R	R	R	R	R	R	S	S	R	R	R	R	R	R	R	R	S	S	S	yes
K051	NO																						

K052	YES	S	R	R	R	R	R	R	R	R	S	S	R	R	R	R	S	R	R	R	R	R	R	yes
K053	YES	R	R	R	I	R	R	R	R	R	S	S	I	I	R	R	R	R	R	R	R	R	R	yes
K054	NO																							
K055	YES	S	R	R	S	R	R	R	S	R	R	R	R	R	R	R	S	R	R	R	R	R	S	yes
K056	NO																							
K057	YES	S	S	S	S	S	S	S	S	S	S	S	S	S	S	R	R	R	S	R	S	S	S	no
K058	YES	S	R	S	I	S	R	R	S	S	S	S	S	S	S	R	R	R	R	R	S	S	R	no
K059	NO																							
K060	NO																							
K061	NO																							
K062	YES	S	S	S	S	S	S	S	S	S	S	S	S	S	S	R	R	R	R	R	S	R	R	no
K063	NO																							
K064	NO																							
K065	YES	S	R	S	R	R	R	R	S	R	S	S	R	R	R	R	S	R	R	R	R	R	R	yes
K066	YES	S	R	R	R	R	R	R	R	R	S	S	S	S	S	R	R	R	S	R	R	R	R	yes
K067	NO																							
K068	NO																							
K069	NO																							
K070	NO																							
K071	NO																							
K072	NO																							
K073	NO																							
K074	NO																							
K075	YES	S	R	R	S	S	R	R	S	R	S	S	R	R	R	R	R	R	R	R	S	S	S	no
K076	NO																							
K077	NO																							
K078	NO																							

K079	NO																							
K080	NO																							
K081	NO																							
K082	NO																							
K083	YES	S	R	R	R	R	R	R	R	S	S	S	R	R	R	R	R	R	R	R	S	R	R	yes
K084	YES	S	R	R	S	S	S	S	S	R	S	S	R	R	R	R	R	R	R	R	S	S	S	no
K085	NO																							
K086	YES	S	R	R	R	S	R	R	S	R	R	S	S	S	R	R	R	R	R	R	S	S	R	yes
K087	YES	S	S	S	S	R	S	S	R	R	S	S	S	S	S	R	R	S	R	R	S	S	S	no
K088	NO																							
K089	NO																							
K090	YES	S	R	R	R	S	R	R	S	S	S	S	S	S	R	S	R	R	R	S	S	S	S	yes
K091	YES	S	R	R	R	R	R	R	S	S	S	S	R	R	R	R	R	R	R	R	S	R	R	yes
K092	NO																							
K093	YES	S	R	R	R	R	R	R	S	S	S	S	R	R	R	R	R	R	R	R	R	R	R	yes
K094	NO																							
K095	YES	S	R	R	R	R	R	R	S	S	S	S	S	S	S	R	R	R	R	R	S	S	S	no
K096	NO																							
K097	YES	S	R	R	S	S	S	S	S	S	S	S	S	S	S	R	R	R	R	R	S	S	S	no
K098	NO																							
K099	YES	S	R	R	R	R	R	R	S	S	S	S	S	S	R	R	R	R	R	R	S	S	R	yes
K100	YES	S	R	I	S	S	R	R	S	S	S	S	S	S	S	S	R	R	R	R	S	S	R	yes
K101	NO																							
K102	YES	S	S	S	S	S	S	S	S	R	S	S	S	S	S	S	S	S	S	I	S	S	S	no
K103	YES	S	R	R	S	S	R	R	R	R	S	S	S	S	R	R	R	R	R	R	R	R	S	yes
K104	NO																							
K105	NO																							

K106	NO																							
K107	NO																							
K108	YES	S	R	S	R	S	R	R	S	R	S	S	R	R	R	S	S	R	R	R	S	R	S	yes
K109	NO																							
K110	YES	S	S	S	S	S	S	S	S	R	S	S	S	S	S	R	S	R	R	R	S	R	R	yes
K111	YES	S	R	R	S	S	S	S	S	S	S	S	R	R	R	R	R	R	S	R	S	R	R	yes
K112	YES	S	R	R	S	S	R	R	S	S	S	S	R	R	R	R	R	R	R	R	S	S	S	yes
K113	NO																							
K114	NO																							
K115	YES	S	S	S	S	S	R	S	S	S	S	S	R	R	R	S	R	R	R	R	S	S	S	yes
K116	NO																							
K117	YES	S	R	R	R	S	R	R	R	S	R	S	S	S	S	R	R	R	R	R	S	S	S	yes
K118	YES	S	R	R	R	R	R	R	S	R	S	S	R	R	R	R	R	R	R	R	S	R	S	yes
K119	NO																							
K120	NO																							
K121	NO																							
K122	NO																							
K123	NO																							
K124	NO																							
K125	NO																							
K126	YES	S	R	R	S	S	S	S	S	R	S	S	R	R	R	R	S	R	R	R	S	R	S	yes
K127	NO																							
K128	YES	S	R	S	R	R	R	S	S	R	S	S	S	S	R	R	R	R	R	R	S	R	S	yes
K129	NO																							
K130	NO																							
K131	YES	S	R	R	R	R	R	R	S	R	I	S	R	R	R	R	S	R	R	R	S	R	R	yes
K132	YES	S	R	R	R	S	R	R	S	R	S	S	R	R	R	R	R	R	R	R	S	R	S	yes

K133	NO																							
K134	NO																							
K135	NO																							
K136	YES	S	R	I	S	S	S	R	S	R	S	S	S	S	R	R	S	R	R	R	S	S	S	yes
K137	NO																							
K138	NO																							
K139	YES	S	R	I	R	R	R	R	S	R	S	S	R	R	R	S	R	R	R	R	S	S	S	yes
K140	YES	S	R	S	R	S	R	R	S	R	S	S	I	I	R	R	S	R	S	R	S	S	R	no
K141	NO																							
K142	YES	R	R	R	R	S	S	R	R	R	S	R	S	I	R	R	R	R	R	R	S	R	R	yes
K143	YES	R	R	R	R	R	R	R	R	R	R	R	I	I	R	R	R	R	R	R	R	R	I	yes
K144	YES	S	R	S	R	R	R	R	S	R	R	S	S	S	S	S	R	R	R	R	S	S	S	yes
K145	NO																							
K146	NO																							
K147	NO																							
K148	NO																							
K149	NO																							
K150	YES	S	R	R	R	R	R	R	S	R	R	R	R	R	R	R	R	R	R	R	S	R	S	yes
K151	YES	S	R	S	R	R	R	R	S	R	R	S	S	S	S	R	R	R	R	R	S	S	S	yes
K152	NO																							
K153	NO																							
K154	NO																							
K155	YES	S	R	S	R	R	R	R	S	R	R	S	S	S	S	S	R	R	S	R	S	S	S	yes
K156	NO																							
K157	YES	S	R	R	S	R	R	R	R	R	S	S	S	S	S	R	S	R	R	R	S	S	S	no
K158	NO																							
K159	YES	S	R	R	R	R	S	R	S	R	S	S	S	S	S	R	R	R	R	R	R	R	R	yes

K160	YES	S	R	R	R	R	R	R	S	R	S	S	R	R	R	R	R	R	R	R	R	R	R	yes
K161	NO																							
K162	NO																							
K163	NO																							
K164	YES	S	R	S	R	R	R	R	S	R	R	S	S	S	S	S	R	R	R	R	S	S	S	yes
K165	YES	S	R	R	R	R	R	R	R	R	S	S	S	R	S	R	S	R	R	R	S	S	S	no
K166	NO																							
K167	NO																							
K168	NO																							
K169	NO																							
K170	NO																							
K171	NO																							
K172	NO																							
K173	YES	S	R	R	R	R	R	R	S	R	S	S	R	R	R	R	R	R	R	R	I	R	R	yes
K174	YES	S	R	R	R	R	R	R	S	R	S	S	R	R	R	R	R	R	R	R	R	R	R	yes
K175	YES	S	R	R	R	R	R	R	S	R	S	S	S	S	R	S	R	R	R	R	R	S	S	yes
K176	YES	S	R	R	R	R	R	R	S	R	S	S	R	R	R	R	R	R	R	R	S	R	R	yes
K177	NO																							
K178	YES	S	R	S	R	R	R	R	S	R	S	S	S	S	S	R	R	R	R	R	S	S	S	no
K179	NO																							
K180	NO																							
K181	NO																							
K182	NO																							
K183	NO																							
K184	YES	S	S	S	R	S	S	R	S	R	S	S	S	S	R	S	S	S	S	S	S	S	S	no
K185	NO																							
K186	NO																							

K187	NO																							
K188	NO																							
K189	YES	S	R	R	R	R	R	R	R	R	S	S	R	R	R	R	S	R	R	R	R	R	R	yes
K190	YES	S	R	R	R	R	R	R	S	R	S	S	S	S	R	R	S	R	R	R	R	S	S	yes
K191	YES	S	R	R	I	R	S	R	S	R	S	S	R	R	R	R	R	R	R	R	R	S	S	yes
K192	NO																							
K193	YES	S	R	R	R	R	R	R	S	R	S	S	R	R	R	I	R	R	R	R	S	R	R	yes
K194	NO																							
K195	YES	S	R	R	I	R	R	R	R	R	S	S	S	S	S	R	S	R	R	R	R	R	R	yes
K196	YES	S	S	I	S	R	R	R	S	R	S	S	S	S	S	S	S	R	R	R	S	R	S	no
K197	YES	S	R	S	S	S	S	S	S	S	S	S	S	S	S	S	S	R	S	S	S	S	S	no
K198	NO																							
K199	YES	S	R	R	R	R	R	R	R	R	S	S	S	S	R	R	R	R	R	R	R	R	R	yes
K200	YES	S	R	S	S	S	S	R	S	R	S	S	S	R	S	R	R	R	R	R	S	S	R	yes
K201	YES	S	R	R	R	R	R	R	S	R	S	S	R	R	R	S	S	R	R	R	R	R	R	yes
K202	NO																							
K203	YES	S	R	S	R	S	R	R	S	R	S	S	S	S	R	R	S	R	R	R	S	S	S	no
K204	NO																							
K205	NO																							
K206	NO	S	S	S	S	S	S	R	R	R	S	S	S	S	R	R	S	R	R	R	S	R	S	yes
K207	YES																							
K208	NO																							
K209	NO																							
K210	NO																							
K211	NO																							
K212	NO																							
K213	NO																							

K214	NO																							
K215	NO																							
K216	NO																							
K217	NO																							
K218	NO																							
K219	NO																							
K220	NO																							
K221	YES	S	S	S	S	S	R	S	S	S	S	S	S	S	S	S	S	S	S	S	S	S	S	no
K222	NO																							
K223	NO																							
K224	NO																							
K225	NO																							
K226	NO																							
K227	NO																							
K228	NO																							
K229	NO																							
K230	NO																							
K231	NO																							
K232	NO																							
K233	YES	S	S	S	R	S	S	S	S	S	S	S	S	S	S	S	S	R	S	R	S	S	S	no
K234	NO																							
K235	NO																							
K236	NO																							
K237	NO																							
K238	NO																							
K239	NO																							
K240	NO																							

K241	NO																							
K242	NO																							
K243	NO																							
K244	NO																							
K245	NO																							
K246	NO																							
K247	YES	S	R	R	R	R	R	R	R	R	S	S	R	R	R	R	R	R	R	R	S	R	R	yes
K248	NO																							
K249	YES	S	R	S	R	R	S	R	S	R	S	S	S	S	S	S	S	R	R	R	S	S	S	no
K250	YES	S	R	R	R	S	R	R	S	R	S	S	R	R	R	R	R	R	R	R	S	R	R	yes
K251	NO																							
K252	YES	S	R	R	R	R	R	R	S	R	S	S	R	R	R	R	R	R	R	R	R	R	R	yes
K253	NO																							
K254	YES	S	S	S	R	R	R	R	S	R	S	S	S	S	R	S	S	R	R	R	S	S	S	no
K255	NO																							
K256	YES	S	R	S	R	R	R	R	S	R	S	S	S	S	S	S	S	R	R	R	I	S	S	no
K257	YES	S	R	S	R	R	R	R	R	R	S	S	R	R	R	R	R	R	R	R	S	S	S	yes
K258	NO																							
K259	NO																							
K260	YES	S	S	S	R	R	R	R	S	R	S	S	S	S	S	S	S	R	R	R	S	S	S	no
K261	YES	S	R	R	R	R	R	R	R	R	S	S	R	R	R	R	R	R	R	R	R	R	R	yes
K262	NO																							
K263	YES	S	R	R	R	R	R	R	R	R	S	S	R	R	R	S	S	R	R	R	S	S	S	yes
K264	NO																							
K265	NO																							
K266	NO																							
K267	YES	S	R	R	R	R	R	R	S	R	S	S	R	R	R	R	R	R	R	R	R	R	R	yes

K268	NO																							
K269	NO																							
K270	YES	S	R	S	R	R	R	R	S	R	S	S	S	S	S	S	S	R	R	R	I	S	S	no
K271	NO																							
K272	YES	S	R	I	S	R	S	R	R	R	S	S	S	S	S	S	S	R	R	R	R	R	S	yes
K273	NO																							
K274	NO																							
K275	NO																							
K276	NO																							
K277	NO																							
K278	YES	S	R	R	R	R	R	R	I	R	S	S	S	S	S	S	S	R	R	R	R	R	R	yes
K279	YES	S	R	R	R	R	S	R	S	R	S	S	S	S	S	S	S	R	R	R	S	S	R	no
K280	YES	S	R	R	R	R	R	R	R	R	S	S	R	R	R	R	R	R	R	R	S	R	S	yes
K281	YES	S	R	S	R	R	R	R	S	R	S	S	I	I	S	S	S	R	R	R	I	S	S	no
K282	YES	S	R	R	R	R	R	R	S	R	S	S	S	S	R	R	R	R	R	R	R	S	S	yes
K283	NO																							
K284	YES	S	R	R	R	R	R	R	S	R	S	S	S	S	R	R	R	R	R	R	S	S	S	yes
K285	NO																							
K286	YES	S	R	R	R	R	R	R	R	R	S	S	R	R	R	R	R	R	R	R	R	I	R	yes
K287	YES	S	R	R	R	R	R	R	R	R	S	S	R	R	R	R	S	R	R	R	S	S	S	no
K288	NO																							
K289	NO																							
K290	NO																							
K291	NO																							
K292	YES	S	R	S	R	R	R	R	S	R	S	S	S	S	R	S	S	R	R	R	S	S	S	no
K293	YES	S	R	S	S	R	S	R	R	R	S	S	S	S	S	S	S	R	R	R	S	S	S	yes
K294	NO																							

K295	NO																							
K296	NO																							
K297	NO																							
K298	NO																							
K299	NO																							
K300	NO																							
K301	NO																							
K302	NO																							
K303	NO																							
K304	NO																							
K305	NO																							
K306	NO																							
K307	NO																							
D001	YES	S	R	S	S	S	R	R	S	S	S	S	S	S	S	R	S	R	R	R	S	S	S	no
D002	YES	S	R	S	S	S	S	S	S	S	S	S	R	R	R	S	S	R	R	R	S	S	S	no
D003	YES	S	S	S	S	S	S	S	S	S	S	S	S	S	S	S	S	S	S	S	S	S	S	no
D004	YES	S	R	R	R	R	R	R	R	R	S	S	R	R	R	R	S	R	R	R	S	S	S	yes
D005	YES	S	R	S	S	S	S	S	S	R	S	S	S	S	S	S	S	S	S	R	S	S	S	no
D006	YES	S	S	S	S	S	S	R	S	S	S	S	S	S	S	S	S	S	R	S	S	S	S	no
D007	YES	S	R	S	S	R	R	R	S	R	S	S	R	R	R	R	R	R	R	R	S	S	S	yes
D008	YES	S	S	S	S	S	S	S	S	S	S	S	S	S	S	S	S	S	S	S	S	R	S	no
D009	YES	S	R	R	R	R	R	R	S	S	S	S	S	S	R	R	R	R	R	R	S	S	S	yes
D010	YES	S	S	S	S	S	S	S	S	S	S	S	S	S	S	S	S	S	S	S	S	S	S	no
D011	YES	S	R	S	S	S	S	S	S	S	S	S	S	S	S	S	S	S	S	R	S	S	S	no
D012	YES	S	R	S	R	R	R	R	R	R	R	S	R	R	S	R	R	R	R	R	S	R	S	yes
D013	YES	S	R	R	R	R	R	R	R	R	S	S	R	R	R	R	R	R	S	R	R	R	S	yes
D014	NO																							

D015	YES	S	R	S	R	R	R	I	R	S	S	S	S	S	S	S	S	R	R	R	S	S	S	no
D016	YES	S	R	S	S	S	S	S	S	S	S	S	S	S	R	S	S	R	S	R	S	S	S	no
D017	YES	S	R	S	S	S	S	S	S	S	S	S	S	S	S	S	S	S	R	R	S	S	S	no
D018	NO																							
D019	YES	S	S	R	S	S	S	S	S	S	S	R	S	S	S	S	S	S	S	S	S	S	S	no
D020	YES	S	R	S	S	S	S	S	R	R	S	S	R	S	R	S	R	R	R	R	R	S	S	no
D021	NO																							
D022	NO																							
D023	YES	R	R	R	R	R	R	R	R	R	S	S	R	R	R	R	R	R	S	R	R	S	R	yes
D024	YES	S	S	R	S	R	R	R	R	R	R	S	S	R	S	S	R	R	R	R	S	S	S	yes
D025	YES	S	S	S	S	S	S	S	S	S	S	S	S	S	S	S	S	S	S	S	S	S	S	no
D026	YES	S	R	S	S	S	S	S	S	S	S	S	S	S	R	S	S	R	S	S	S	S	S	no
D027	YES	S	S	S	S	S	S	S	S	S	S	S	S	S	S	S	R	S	R	S	S	S	S	no
D028	YES	S	R	S	S	R	R	R	R	R	S	S	R	R	R	R	S	R	R	R	S	S	S	yes
D029	YES	S	R	R	S	S	S	S	R	R	S	S	S	S	R	R	I	S	R	R	R	I	S	yes
D030	YES	S	R	R	S	R	R	R	S	R	S	S	R	R	R	S	S	R	R	S	S	S	S	yes
D031	NO																							
D032	NO																							
D033	YES	S	S	S	S	S	S	S	S	S	S	S	S	S	S	S	S	S	S	S	S	S	S	no
D034	YES	S	R	R	R	R	R	R	R	R	S	S	R	R	R	R	R	R	R	R	R	R	S	yes
D035	YES	S	R	R	R	R	R	R	S	R	S	S	R	R	R	R	R	R	S	R	R	R	S	yes
D036	YES	S	R	S	S	S	S	S	S	S	S	S	S	S	S	S	S	R	R	S	S	S	S	no
D037	NO																							
D038	YES	S	R	S	S	S	S	S	S	S	S	S	S	S	S	S	S	S	S	S	S	S	S	no
D039	YES	S	R	S	S	S	S	S	S	I	S	S	S	S	S	R	S	S	S	R	S	R	S	no
D040	YES	S	R	R	R	R	R	R	R	R	S	S	R	R	R	R	R	R	R	R	S	S	S	yes
D041	NO																							

D042	YES	S	R	S	S	S	S	S	R	S	S	S	S	S	S	S	S	R	R	S	S	S	S	no
D043	NO																							
D044	NO																							
D045	NO																							
D046	NO																							
D047	YES	S	R	S	S	S	S	S	R	S	S	S	R	R	R	S	S	S	R	S	S	S	S	yes
D048	NO																							
D049	YES	S	R	R	R	R	R	R	S	R	S	S	R	R	R	R	R	R	R	R	S	S	S	yes
D050	YES	S	S	S	R	R	R	R	R	R	S	S	S	S	S	S	R	R	R	R	R	R	S	yes
D051	NO																							
D052	YES	S	R	S	S	S	R	R	R	S	S	S	S	S	S	R	S	S	R	S	S	S	S	yes
D053	NO																							
D054	NO																							
D055	NO																							
D056	YES	S	R	S	R	R	R	R	S	S	S	S	S	S	S	R	S	R	R	R	S	I	S	no
D057	NO																							
D058	NO																							
D059	YES	S	R	S	S	S	S	S	R	S	S	S	S	S	R	S	S	S	R	S	S	S	S	yes
D060	YES	S	R	R	R	R	R	R	R	R	R	R	R	R	R	R	S	R	R	R	R	S	S	yes
D061	YES	S	S	S	S	S	S	S	S	S	S	S	S	S	S	S	S	S	S	S	S	S	S	no
D062	NO																							
D063	NO																							
D064	YES	S	R	S	R	R	R	R	S	R	S	S	R	R	R	R	R	R	R	R	R	R	S	yes
D065	NO																							
D066	YES	S	S	S	S	S	S	S	S	S	S	S	S	S	R	S	S	R	S	S	S	R	S	no
D067	YES	S	R	R	R	R	R	R	R	R	S	S	R	R	R	R	S	R	R	R	S	S	S	yes
D068	NO																							

D069	NO																							
D070	YES	S	S	S	S	S	S	S	S	S	S	S	S	S	S	S	S	S	S	S	S	S	S	no
D071	NO																							
D072	YES	S	R	R	S	S	S	S	R	R	S	S	S	S	S	R	S	R	R	R	S	S	S	yes
D073	YES	S	R	S	S	S	S	S	S	S	S	S	R	R	R	S	S	R	R	S	S	S	S	no
D074	NO																							
D075	YES	S	S	S	S	S	S	S	S	S	S	S	S	S	R	S	S	S	S	S	S	S	S	no
D076	NO																							
D077	YES	S	S	S	S	S	S	S	S	S	S	S	S	S	S	S	S	S	S	S	S	S	S	no
D078	NO																							
D079	YES	S	S	S	S	S	S	S	S	S	S	S	S	S	S	S	S	S	S	S	S	S	S	no
D080	YES	S	S	S	R	S	S	S	S	S	S	S	S	S	S	S	S	S	S	S	S	S	S	no
D081	YES	S	R	S	S	S	S	S	R	R	S	S	R	R	R	S	S	R	R	R	S	S	S	yes
D082	YES	S	S	S	S	S	S	S	S	S	S	S	S	S	S	S	S	S	S	S	S	R	S	no
D083	YES	S	R	S	S	S	S	S	S	S	S	S	S	S	S	S	S	S	R	S	S	S	S	no
D084	NO																							
D085	NO																							
D086	YES	S	R	R	S	R	R	R	S	S	S	S	R	R	R	S	R	R	R	R	S	S	S	yes
D087	NO																							
D088	NO																							
D089	YES	S	R	R	R	R	R	R	S	R	S	S	R	R	R	R	R	R	R	R	R	R	S	yes
D090	YES	S	R	R	R	S	R	R	S	R	S	S	R	R	R	R	S	R	R	R	S	R	S	yes
D091	YES	S	R	S	S	S	R	S	S	S	S	S	S	S	R	R	S	S	R	S	S	S	S	no
D092	YES	S	S	S	S	S	S	S	S	S	S	S	S	S	S	S	S	S	R	S	S	S	S	no
D093	YES	S	S	S	S	S	S	S	R	S	S	S	S	S	S	S	I	S	S	R	S	S	S	no
D094	YES	S	S	R	S	R	R	R	R	S	S	S	R	R	R	S	S	R	R	R	S	S	S	no
D095	YES	S	S	S	R	R	R	R	S	S	S	S	S	S	R	S	R	S	R	R	R	R	S	yes

D096	YES	S	R	R	S	S	S	S	S	S	S	S	S	S	S	S	S	R	S	S	S	S	S	no
D097	YES	S	R	S	S	S	S	S	S	S	S	S	S	S	R	S	S	R	R	S	S	S	S	no
D098	YES	S	R	R	R	R	R	R	S	S	S	S	S	R	R	R	R	R	R	R	R	R	S	yes
D099	YES	S	R	R	R	R	R	R	R	S	S	S	R	R	R	R	R	R	R	R	R	R	S	yes
D100	NO																							
D101	NO																							
D102	YES	S	R	S	R	S	S	S	S	S	S	S	S	S	S	R	S	R	R	S	S	S	S	no
D103	NO																							
D104	NO																							
D105	NO																							
D106	YES	S	S	S	R	S	S	S	S	R	S	S	R	R	R	S	S	S	R	R	S	S	S	yes
D107	YES	S	R	S	S	R	S	S	I	I	S	S	R	R	S	R	R	I	R	R	I	I	S	no
D108	NO																							
D109	NO																							
D110	NO																							
D111	NO																							
D112	YES	S	R	R	R	R	R	R	R	R	S	S	S	S	S	R	R	R	R	R	R	R	S	no
D113	NO																							
D114	NO																							
D115	NO																							
D116	NO																							
D117	NO																							
D118	NO																							
D119	NO																							
D120	YES	S	R	S	S	S	S	S	S	S	S	S	R	R	R	S	S	R	R	S	S	S	S	no
D121	NO																							
D122	YES	S	S	S	S	S	S	S	S	S	S	S	S	S	S	S	S	S	S	S	S	S	S	no

D123	NO																							
D124	NO																							
D125	YES	R	R	S	S	R	R	R	S	R	S	S	S	S	R	R	S	R	R	R	S	S	S	yes
D126	NO																							
D127	NO																							
D128	NO																							
D129	NO																							
D130	NO																							
D131	YES	S	R	S	S	S	R	R	S	S	S	S	S	S	R	S	S	R	S	S	S	S	R	no
D132	NO																							
D133	NO																							
D134	NO																							
D135	NO																							
D136	NO																							
D137	NO																							
D138	YES	S	R	S	S	R	R	R	S	S	S	S	S	S	S	S	S	S	R	S	S	S	S	no
D139	NO																							
D140	NO																							
D141	NO																							
D142	NO																							
D143	NO																							
D144	NO																							
D145	YES	S	S	S	S	S	S	S	S	S	S	S	S	S	R	S	S	S	R	S	S	S	S	no
D146	NO																							
D147	NO																							
D148	NO																							
D149	NO																							

D150	NO																							
D151	NO																							
D152	NO																							
D153	YES	S	S	S	S	S	S	S	S	S	S	S	S	S	S	S	S	S	R	S	S	S	S	no
D154	YES	S	R	S	S	R	R	R	S	S	S	S	S	S	S	S	S	S	R	S	S	S	S	no
D155	NO																							
D156	NO																							
D157	NO																							
D158	NO																							
D159	NO																							
D160	NO																							
D161	NO																							
D162	NO																							
D163	NO																							
D164	NO																							
D165	YES	S	R	S	S	S	S	S	S	S	S	S	S	S	R	S	S	S	S	S	S	S	S	no
D166	NO																							
D167	NO																							
D168	NO																							
D169	NO																							
D170	NO																							
D171	YES	S	R	S	S	S	R	S	S	S	S	S	S	S	S	R	S	R	S	S	S	S	S	no
D172	YES	S	R	S	S	R	R	R	S	S	S	S	S	S	S	S	S	S	R	S	S	S	S	no
D173	NO																							
D174	NO																							
D175	NO																							
D176	NO																							

D177	NO																							
D178	NO																							
D179	NO																							
D180	YES	S	S	S	S	S	S	S	S	S	S	S	S	S	S	S	S	S	S	S	S	S	S	no
D181	YES	S	S	S	S	S	S	S	S	S	S	S	S	S	R	S	S	S	R	S	S	S	S	no
D182	NO																							
D183	NO																							
D184	NO																							
D185	YES	S	S	S	S	S	S	S	S	S	S	S	S	S	S	S	S	S	S	S	S	S	S	no
D186	NO																							
D187	YES	S	R	S	S	S	S	S	S	S	S	S	S	S	R	S	S	S	S	S	S	S	S	no
D188	YES	S	R	R	S	R	S	S	S	S	S	S	S	S	R	S	S	R	R	S	S	S	S	no
D189	NO																							
D190	NO																							
D191	NO																							
D192	NO																							
D193	NO																							
D194	NO																							
D195	NO																							
D196	NO																							
D197	NO																							
D198	NO																							
D199	NO																							
D200	NO																							MDR

Legends : IPM: Imipenem, AMX: Amoxicillin, AMC: Amoxiclav, CAZ: Ceftazidim, FOX: Cefoxitin, CTX: Cefotaxim, CXM: Cefuroxim, PRL: Piperacillin, CTR: Ceftriazone AMK: Amikacin, GEN: Gentamicin, CIP: Ciprofloxacin, OFX: Ofloxacin, NAL: Naxidilic acid, COT: Trimethoprim-sulfamethoxazole, COL: Colistin, TET: Tetracyclin, VAN: vancomycin NIT: Nitrofurantoin, ATM: Aztreonam, FOS: Fosfomycin, ERY: Erythromycin, S: Sensitive, I: Intermediate, R: Resistant, MDR Multidrug resistant

S3. Detection of virulent factors and resistance genes

Code	**ESBL gene**	bla_{TEM}	bla_{OXA}	bla_{SHV}	$bla_{CTX\text{-}M}$	$bla_{CTX\text{-}M}$ + bla_{SHV}	$bla_{CTX\text{-}M}$ + bla_{OXA}	bla_{TEM} + $bla_{CTX\text{–}M}$	bla_{TEM} + bla_{OXA}	bla_{TEM} + bla_{SHV}	bfpA/EPEC	AggR/EAEC	LT/ETEC	stx/STEC	Ipah/EIEC	*SEROVAR*
K001																
K002																
K003	yes	yes	yes	yes	yes	yes	yes	yes	yes	yes	no	no	no	no	yes	*Enteroinvasive E. coli*
K039																
K040	yes	yes	no	yes	no	no	no	no	no	yes	no	no	no	no	yes	*Enteroinvasive E. coli*
K041																
K042	yes	yes	no	no	yes	no	no	yes	no	no	yes	no	no	no	no	*Enteropathogenic E. coli*
K043																
K073																
K074																
K075	yes	yes	no	no	yes	no	no	yes	no	no	no	no	yes	no	no	*Enterotoxigenic E. coli*
K076																
K077																
K078																
K079																
K080																
K081																
K082																
K083	yes	yes	no	no	yes	no	no	yes	no	no	no	no	yes	no	no	*Enterotoxigenic E. coli*
K084																
K085																
K086																

K087	yes	no	no	yes	no	no	no	no	no	no	yes	no	no	no	no	*Enteropathogenic E. coli*
K088																
K089																
K090																
K091	yes	yes	yes	no	no	no	no	no	yes	no	yes	no	no	no	no	*Enteropathogenic E. coli*
K092																
K108																
K109																
K110	yes	yes	no	no	yes	no	no	yes	no	no	yes	no	no	no	no	*Enteropathogenic E. coli*
K111																
K112	yes	yes	no	no	no	no	no	no	no	no	yes	no	no	no	no	*Enteropathogenic E. coli*
K113																
K125																
K126	yes	yes	yes	no	yes	no	yes	yes	yes	no	yes	no	no	no	no	*Enteropathogenic E. coli*
K127																
K142																
K143	yes	yes	yes	yes	yes	yes	yes	yes	yes	yes	yes	no	no	no	no	*Enteropathogenic E. coli*
K144	yes	yes	yes	yes	yes	yes	yes	yes	yes	yes	no	no	yes	no	no	*Enterotoxigenic E. coli*
K145																
K146																
K147																
K148																
K149																
K150	yes	yes	yes	yes	yes	yes	yes	yes	yes	yes	no	no	no	yes	no	*Shiga-toxin E coli*

K151																
K152																
K153																
K154																
K155																
K156																
K157	yes	yes	no	no	yes	no	no	yes	no	no	no	no	no	yes	no	*Shiga-toxin E coli*
K158																
K159	yes	yes	no	yes	yes	yes	no	yes	no	yes	yes	no	no	no	no	*Enteropathogenic E. coli*
K160	yes	yes	no	yes	yes	yes	no	yes	no	yes	yes	no	no	no	no	*Enteropathogenic E. coli*
K161																
K162																
K163																
K164	yes	yes	no	yes	yes	yes	no	yes	no	yes	no	yes	no	no	no	*Enteroaggregative E. coli.*
K165	yes	yes	no	yes	yes	yes	no	yes	no	yes	no	yes	no	no	no	*Enteroaggregative E. coli.*
K166																
K173	yes	yes	yes	yes	no	no	no	no	yes	yes	no	no	no	yes	no	*Shiga-toxin E coli*
K174																
K175	yes	yes	yes	yes	yes	yes	yes	yes	yes	yes	no	yes	no	no	no	*Enteroaggregative E. coli.*
K176																
K177																
K178	yes	no	no	yes	yes	yes	no	no	no	no	yes	no	no	no	no	*Enteropathogenic E. coli*
K179																
K189	yes	yes	yes	no	yes	no	yes	yes	yes	no	no	yes	no	no	no	*Enteroaggregative E. coli.*
K190																

K191																
K192																
K193																
K194																
K19 5	yes	yes	no	yes	no	no	no	no	no	yes	yes	no	no	no	no	*Enteropathogenic E. coli*
K19 6	yes	yes	no	yes	yes	yes	no	yes	no	yes	yes	no	no	no	no	*Enteropathogenic E. coli*
K197																
K198																
K199																
K200																
K20 1	yes	no	yes	no	yes	no	yes	no	no	no	yes	no	no	no	no	*Enteropathogenic E. coli*
K202																
K203																
K204																
K205																
K20 6	yes	yes	yes	no	yes	no	yes	yes	yes	no	yes	no	no	no	no	*Enteropathogenic E. coli*
K207																
K246																
K24 7	yes	yes	yes	no	yes	no	yes	yes	yes	no	no	yes	no	no	no	*Enteroaggregative E. co li.*
K248																
K249																
K25 0	yes	yes	yes	yes	yes	yes	yes	yes	yes	yes	no	yes	no	no	no	*Enteroaggregative E. co li.*
K251																
K25 2	yes	yes	yes	yes	yes	yes	yes	yes	yes	yes	yes	no	no	no	no	*Enteropathogenic E. coli*
K253																

K262																
K263	yes	yes	yes	yes	yes	yes	yes	yes	yes	yes	no	no	no	yes	no	*Shiga-toxin E coli*
K264																
K265																
K266																
K267	yes	yes	yes	no	yes	no	yes	yes	yes	no	yes	no	no	no	no	*Enteropathogenic E. coli*
K268																
K279																
K280	yes	yes	no	no	yes	no	no	yes	no	no	yes	no	no	no	no	*Enteropathogenic E. coli*
K281																
K282	yes	yes	yes	no	yes	no	yes	yes	yes	no	yes	no	no	no	no	*Enteropathogenic E. coli*
K283																
K284	yes	yes	no	no	yes	no	no	yes	no	no	no	yes	no	no	no	*Enteroaggregative E. coli.*
K285																
K286	yes	yes	yes	no	yes	no	yes	yes	yes	no	no	no	no	yes	no	*Shiga-toxin E coli*
K287																
K288																
K289																
K290																
K291																
K292	yes	yes	yes	no	yes	no	yes	yes	yes	no	no	no	yes	no	no	*Enterotoxigenic E. coli*
K293	yes	yes	yes	no	yes	no	yes	yes	yes	no	no	no	no	yes	no	*Shiga-toxin E coli*
K307																
D001	yes	yes	no	no	no	no	no	no	no	no	no	no	no	yes	no	*Shiga-toxin E coli*
D00	no	no	no	no	no	no	no	no	no	no	yes	no	no	no	no	*Enteropathogenic E. coli*

2															
D003	no	no	no	no	no	no	no	no	no	no	no	yes	no	no	no	*Enteroaggregative E. coli.*
D004	yes	yes	no	no	no	no	no	no	no	no	no	no	no	yes	no	*Shiga-toxin E coli*
D005	yes	yes	no	yes	no	no	no	no	no	yes	no	no	yes	no	no	*Enterotoxigenic E. coli*
D006																
D007																
D008																
D009	yes	no	no	no	yes	no	no	no	no	no	yes	no	no	no	no	*Enteropathogenic E. coli*
D010	no	no	no	no	no	no	no	no	no	no	no	yes	no	no	no	*Enteroaggregative E. coli.*
D022																
D023	yes	yes	yes	no	no	no	no	no	yes	no	yes	no	no	no	no	*Enteropathogenic E. coli*
D033																
D034	yes	yes	no	no	yes	no	no	yes	no	no	yes	no	no	no	no	*Enteropathogenic E. coli*
D035	yes	no	no	no	yes	no	no	no	no	no	yes	no	no	no	no	*Enteropathogenic E. coli*
D036																
D037																
D038																
D039																
D040	yes	no	no	yes	no	no	no	no	no	no	no	yes	no	no	no	*Enteroaggregative E. coli.*
D041																
D048																

D049	yes	yes	yes	no	yes	no	yes	yes	yes	no	yes	no	no	no	no	*Enteropathogenic E. coli*
D050																
D056	yes	no	no	no	yes	no	no	no	no	no	no	no	yes	no	no	*Enterotoxigenic E. coli*
D057																
D058																
D059																
D060	yes	yes	yes	no	no	no	no	no	yes	no	no	yes	no	no	no	*Enteroaggregative E. coli.*
D081	yes	no	yes	no	yes	no	yes	no	no	no	no	yes	no	no	no	*Enteroaggregative E. coli.*
D082																
D097																
D098		yes	no	no	no	no	no	no	no	no	no	no	no	yes	no	*Shiga-toxin E coli*
D099																
D105																
D106	yes	yes	no	no	no	no	no	no	no	no	no	yes	no	no	no	*Enteroaggregative E. coli.*
D107																
D125	yes	yes	no	no	no	no	no	no	no	no	no	no	no	yes	no	*Shiga-toxin E coli*
D126																

S4. Image of the gel showing amplification

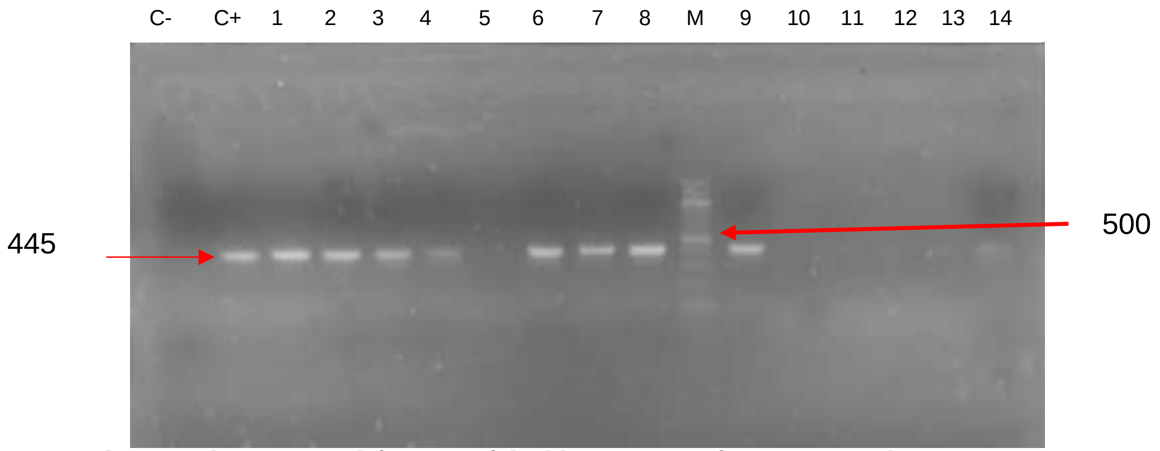

Gel image showing amplification of the blaTEM gene fragment (445 bp). C-: negative control, C+: positive control, M: molecular weight marker (100 bp scale). Lines 1 to 4 and 6 to 8 and 9 are positive isolates containing the blaTEM gene.

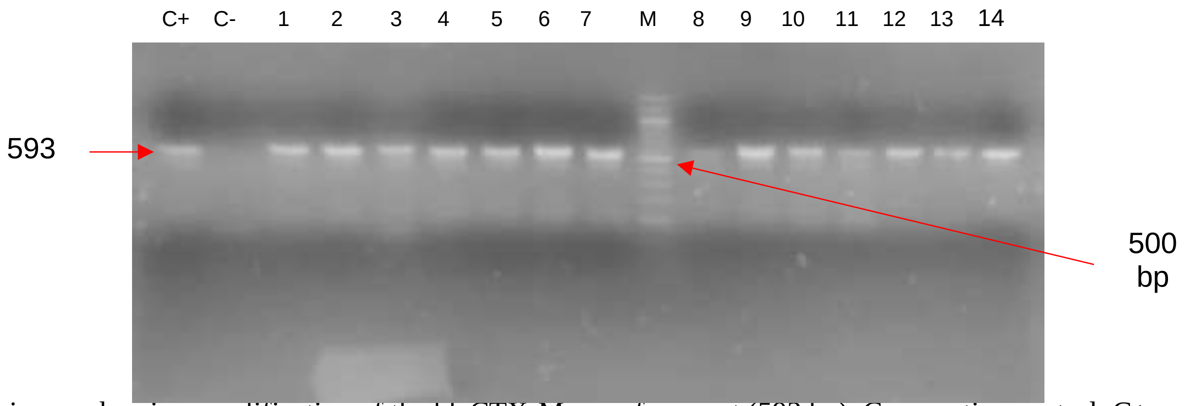

Gel image showing amplification of the blaCTX-M gene fragment (593 bp). C-: negative control, C+: positive control, M: molecular weight marker (100 bp scale). Lines 1 to 14 correspond to positive isolates containing the blaCTX-M gene.

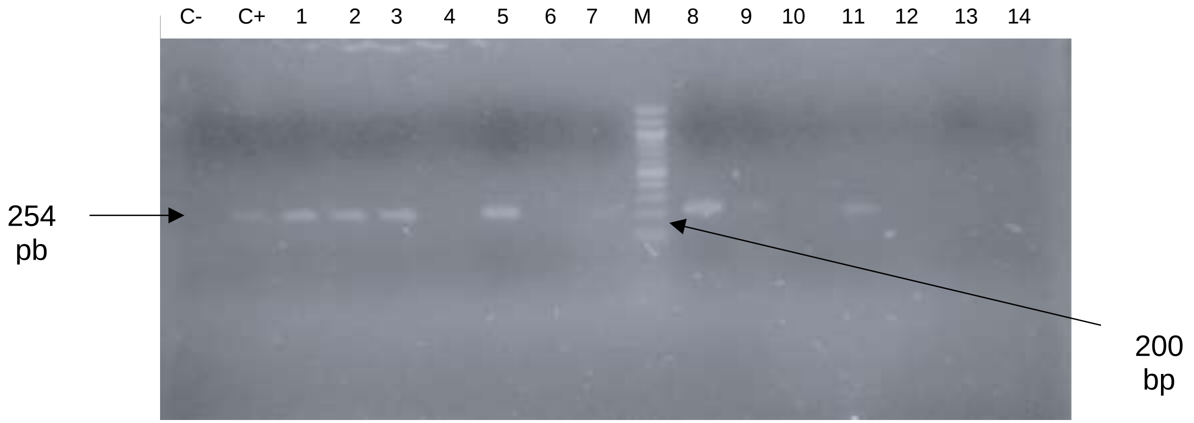

Gel image showing amplification of the AggR virulent gene fragment (254 bp). C-: negative control, C+: positive control, M: molecular weight marker (100 bp scale). Lines 1 to 3, 5, 8, and 11 are positive isolates containing AggR.

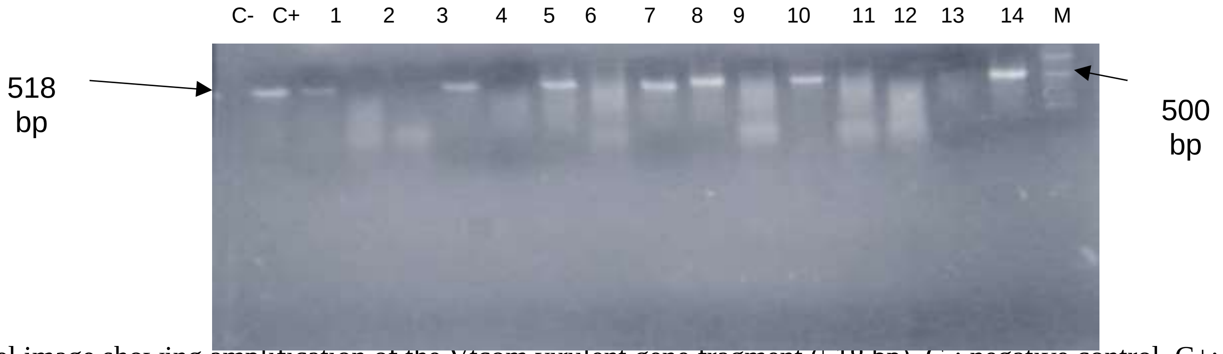

Gel image showing amplification of the Vtcom virulent gene fragment (518 bp). C-: negative control, C+: positive control, M: molecular weight marker (100 bp scale). Lines 1, 3, 5, 7, 8, 10 and 14 are positive isolates containing Vtcom.
